# The immunomodulatory effects of vitamins in cancer

**DOI:** 10.3389/fimmu.2024.1464329

**Published:** 2024-10-07

**Authors:** Camelia Munteanu, Sorin Marian Mârza, Ionel Papuc

**Affiliations:** ^1^ Department of Plant Culture, Faculty of Agriculture, University of Agricultural Sciences and Veterinary Medicine, Cluj-Napoca, ;Romania; ^2^ Faculty of Veterinary Medicine, University of Agricultural Science and Veterinary Medicine, Cluj-Napoca, ;Romania

**Keywords:** cancer, nutrition, fat-soluble vitamins, water-soluble vitamins, immunomodulation cancer, immunity, mammals, vitamins

## Abstract

Nutrition may affect animal health due to the strong link between them. Also, diets improve the healing process in various disease states. Cancer is a disease, where the harmful consequences of tumors severely impair the body. The information regarding the evolution of this disease is extrapolated from human to animal because there are few specific studies regarding nutritional needs in animals with cancer. Thus, this paper aims to review the literature regarding the immunomodulatory effects of vitamins in mammal cancer. An adequate understanding of the metabolism and requirements of nutrients for mammals is essential to ensuring their optimal growth, development, and health, regardless of their food sources. According to these: 1) Some species are highly dependent on vitamin D from food, so special attention must be paid to this aspect. Calcitriol/VDR signaling can activate pro-apoptotic proteins and suppress anti-apoptotic ones. 2) Nitric oxide (NO) production is modulated by vitamin E through inhibiting transcription nuclear factor kappa B (NF-κB) activation. 3) Thiamine supplementation could be responsible for the stimulation of tumor cell proliferation, survival, and resistance to chemotherapy. 4) Also, it was found that the treatment with NO-Cbl in dogs is a viable anti-cancer therapy that capitalizes on the tumor-specific properties of the vitamin B12 receptor. Therefore, diets should contain the appropriate class of compounds in adequate proportions. Also, the limitations of this paper are that some vitamins are intensively studied and at the same time regarding others, there is a lack of information, especially in animals. Therefore, some subsections are longer and more heavily debated than others.

## 1 Introduction

Nutrition, dietary habits, and health are strongly correlated, therefore obtaining an adequate amount of particular nutrients from diet is important. Although the type and quantity of nutrients ingested are closely related to both immune system function and metabolic state, improper nutrient consumption is causally associated with the development of the most important diseases (1). Diet can cause disease in animals and humans, but it can also support the healing process in various disease states.

A special case is cancer, in which the damaging effects of tumors weaken the body. In cancer patients, malnutrition is a prevalent issue that affects 20% to 80% of patients with this disease (2). Unfortunately, malnutrition, including its most severe form, cachexia, is a daily occurrence in hematology and oncology problems. It is caused by the tumor’s effect on the host’s metabolism and the use of increasingly potent cancer treatments. The main impact is a higher probability of complications after long-term radiation therapy, chemotherapy, and/or surgery. Additionally, a serious decline in body status, a significant alteration in the quality of life, and depression that accompanies the patient are all associated with malnutrition (3). Anorexia, often described in both humans as well as companion animals during cancer therapy, is associated with low nutrient intake and can result from either stress, severe pain, or problems in the gastrointestinal (GI) tract (i.e. vomiting as an adverse effect of anti-cancer drugs in dogs and cats). The central nervous system is primarily responsible for modulating the balance of energy, and there is evidence from both human and animal models that changes in this system have a key role in cancer (4). In support of this theory, it was shown that anorexic human cancer patients demonstrated different hypothalamus activity from those without anorexia using functional magnetic resonance imaging (5).

Consequently, it is best to concentrate nutritional approaches to treatment on recognizing the variables and symptoms related to these solid malignancies (6). A decrease in dietary intake is one factor contributing to malnutrition from cancer. Thus, anorexia is linked to inadequate dietary intake and can exacerbate the side effects of cancer treatments. It is commonly characterized by changes in taste, loss of appetite, and feelings of depression. In addition, Tumor necrosis factor- α (TNF α), interleukin (IL)-1, and IL-6 are examples of inflammatory mediators that are released during malnutrition associated with cancer and can control hunger and the body’s absorption of nutrients. Management of this syndrome may extend survival durations and enhance the quality of life for patients diagnosed with related ailments if weight loss/cachexia affects survival.

Regarding animals, inappetence is one of the primary clinical indicators that most veterinary oncologists (98%) cite as being important for classifying canine lymphoma patients as being in substage b, which is linked to a worse clinical prognosis (the median decrease in appetite required to classify the patient as substage b in this survey was approximately 40%) (7). In the case of cats, anorexia may appear within one to two days or may last for a long time. As the frequency of hypoxic or anorexic episodes increase, the concerns about the cat’s nutritional status are increased.

**Figure 1:**
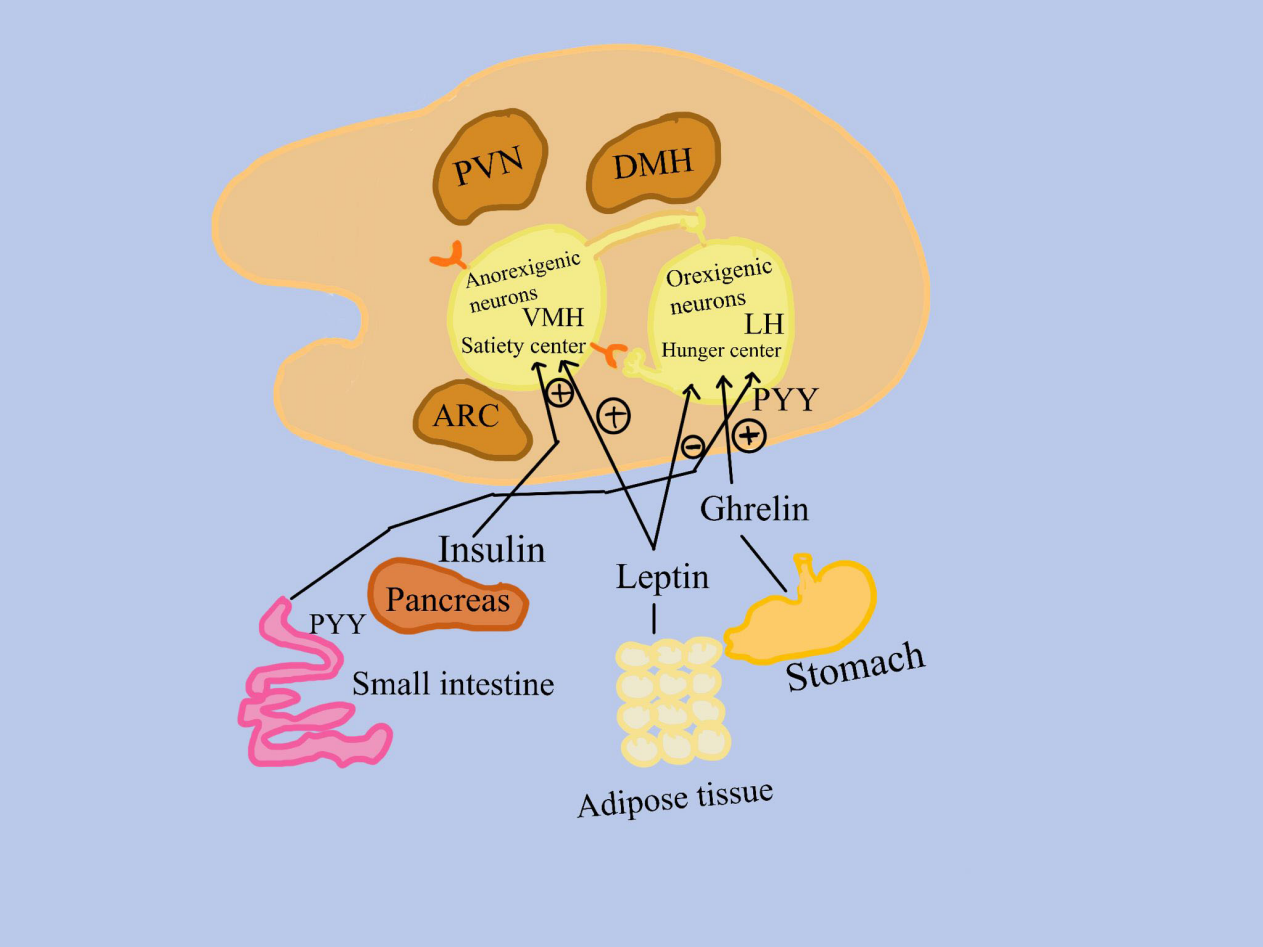
Signals from the peripheral, which includes the gastrointestinal system, adipose tissue, and the pancreas, cause differences in the activity of two subpopulations of neurons. 1. Anorexigenic neurons in the ventromedial hypothalamus activate a satiety center; 2. Orexigenic neurons, found in the lateral hypothalamus, promote appetite. Insulin from the pancreas, peptide tyrosine (PYY) from the small intestine, leptin from adipose tissue, and ghrelin from the stomach bind to receptors on orexigenic and/or anorexigenic neurons in the nuclei of the hypothalamus. LH, the lateral hypothalamus; PVN, the paraventricular hypothalamus; VMH, the ventromedial hypothalamus; ARC, the arcuate nucleus of the hypothalamus; (+), stimulation (–), inhibition.

The hypothalamus is the main organ responsible for controlling hunger for mammals (**Figure 1**). Two neuronal subpopulations present modifications in activity due to signals from the periphery, which includes the pancreas, adipose tissue, and the gastrointestinal tract (8).

One of these populations is represented by appetite-suppressing neurons, which function in these ways: 1. Anorexigenic (appetite-suppressing) neurons activate a satiety center in the ventromedial hypothalamus; 2. Many neurons signal the consequences of eating less and metabolizing more on muscle, adipose tissue, liver, and other tissues; 3. Positive effects are attributed to circulating insulin and leptin. 4. Anorexigenic and orexigenic neurons communicate with each other.

The second population is represented by neurons that stimulate appetite, orexigenic neurons: 1. Trigger the lateral hypothalamic hunger center; 2. show the consequences of eating excess simultaneously with a decrease of metabolization, on muscle, adipose tissue, liver, and other tissues; 3. Favorably impacted by ghrelin, which is mostly produced in the stomach fundus’s oxyntic glands; 4. Insulin, leptin, and circulating peptide YY have a negative effect; 5. Anorexigenic and orexigenic neurons communicate with one another (9, 10) (**Figure 1**).

As previously mentioned, ghrelin is the only known mediator that has a stimulatory effect on orexigenic neurons, whereas many other peripheral signals negatively influence appetite. Ghrelin is therefore thought to be the primary regulator of feeding behavior start, hunger stimulation, and subsequent food intake (10). Growth hormone-releasing peptide 6 (GHRP-6) was discovered to promote the release of growth hormone (GH) from the pituitary gland through a new receptor (GH secretagogue receptor 1a (GHS-R1a), leading to the discovery of ghrelin receptor agonists (GRAs) in the late 1980s. The hypothalamus, pituitary gland, bone, heart, lung, liver, kidney, pancreas, and immune cells have all been shown to express this receptor (9). These discoveries finally resulted in a family of oral small compounds known as GH secretagogues (GHS) or GRAs, which induced the pituitary gland to release growth hormones. It was eventually shown that the natural ligand of GHS-R1a is endogenous ghrelin. GRAs have been shown to have several physiological effects, including stimulation of appetite, GH release from the pituitary gland that leads to an increase in growth factor 1 (IGF-1) *via* the liver, an increase in muscle mass, stimulation of bone formation, improvement of gastrointestinal motility, and anti-inflammatory properties.

The interest in using ghrelin to regulate appetite and the substantial therapeutic problem that inappetence in dogs and cats present made using GRAs for appetite stimulation a logical choice. Important hypothalamic regions linked to feeding behavior, the arcuate and ventromedial nuclei, are locations where GHS-R1a is expressed in humans and other mammals (11). The function of ghrelin in regulating preprandial appetite and the starting point of meal intake has been clarified by studies conducted in various mammals (12). Exogenous ghrelin treatment enhanced the daily food intake of healthy beagle dogs. Research in dogs (13), and cats (14) has demonstrated that ghrelin levels are elevated during fasting and lowered with food consumption. The study of GRAs in cancer anorexia-cachexia syndrome is interesting due to their propensity to stimulate hunger, the increase in IGF-1 observed with GRAs, and the role of IGF-1 in maintaining or hypertrophying muscle mass in dogs (15). A GRA called anamorelin has been investigated for treating cancer-related cachexia in individuals with non-small cell lung cancer (NSCLC) (16).

As mentioned before, nutrition is important in cancer, therefore anticancer diets now include additional essential nutrients conform to the studies on cancer in humans (17) and other mammals. These are represented by vitamin D, certain amino acids and widely used supplements like garlic and turmeric (18–20).

**Figure 2:**
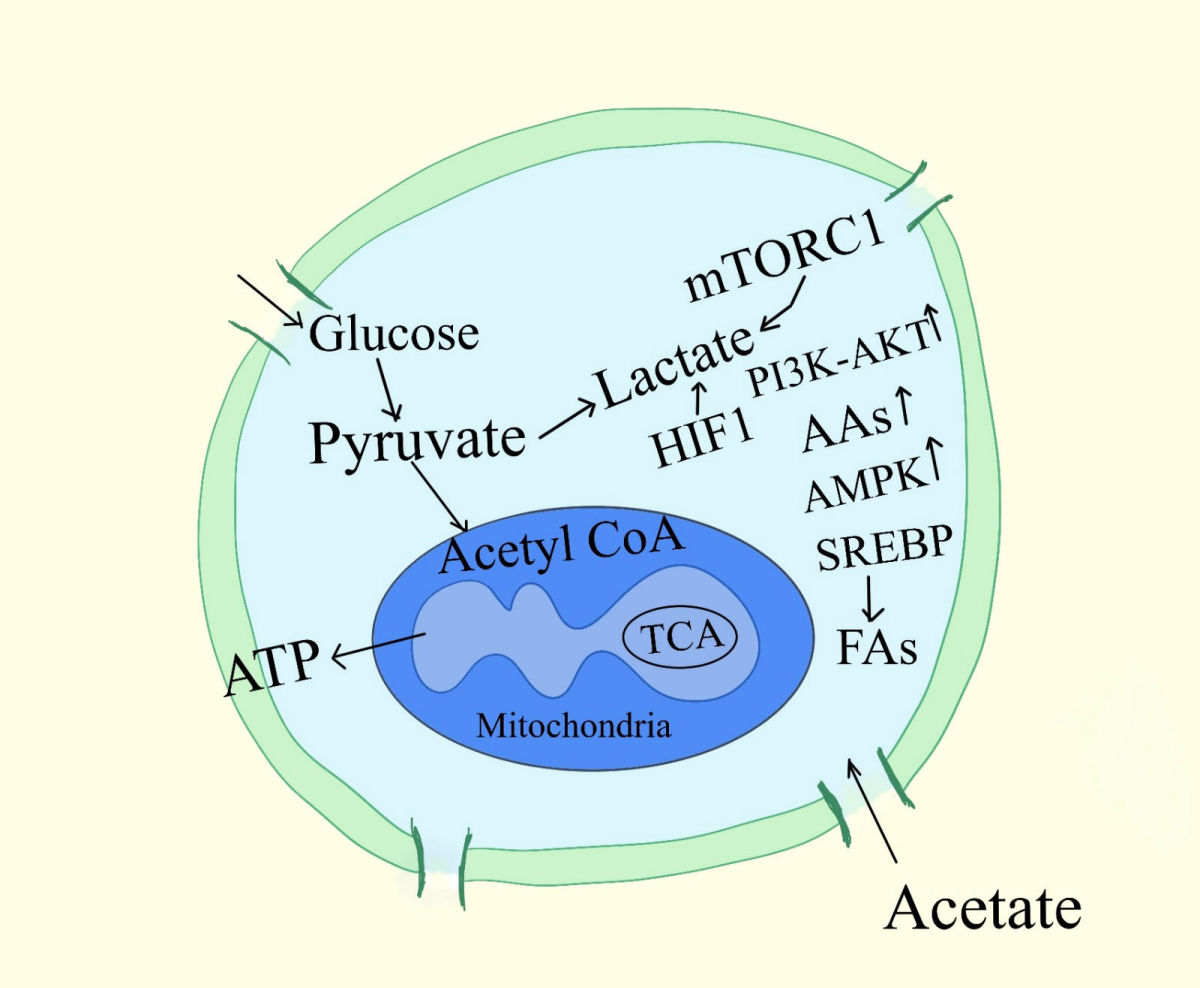
Signaling pathways associated with cancer cell metabolism. A subset of mRNAs considered to stimulate cell growth and proliferation are specifically controlled from being translated by mTORC1, which also regulates the activity of the translational machinery. AAs, amino-acids; G6P, Glucose-6-phosphate, 3-PG, 3-phosphoglycerate, ATP, adenosine 5´-triphosphate, MTORC1, mTOR complex, regulatory-associated protein of mTOR; α-KG, α-ketoglutarate.

Strategies centered on nutrition for reducing the growth of tumors remain uncertain, especially when it comes to carbs. It was discovered more than 60 years ago that tumor cells had a unique capacity to display reduced rates of respiration, which typically cause the death of healthy cells, together with increased glycolysis and pentose phosphate cycle activity (20). The free uptake of glucose by cells, the reduction of cellular mitochondria, leading to anaerobic metabolism for energy to generate lactate in place of pyruvate, and the nutritional concerns for cachexic, frail, sarcopenic pet detoxification of intracellular oxidants are reported to contribute to these tumor cell survival mechanisms (18). As a result of all these, diet guidelines for mammals with cancer suggest limiting their consumption of digestible carbohydrates (**Figure 2**).

Therefore, nutrients have an essential role in the defense function of mammals. The most used nutritional strategy regardless of whether the animals are omnivores or carnivores is the reduction of carbohydrates. The hypothalamus controls all the mechanisms of hunger and satiety in mammals, irrespective of whether they are omnivores or carnivores. It is considered that there is a lack of specialized literature regarding the nutritional requirements in animals with cancer and that most of the information is extrapolated from humans to animals. The absence of specialized literature refers to the lack of biochemical and signaling knowledge mechanisms regarding the immunomodulatory effects in mammals, except for a few species used in laboratory studies. Thus, this paper aims to review the literature regarding the immunomodulatory effects of vitamins in mammal cancer (particularly humans and domestic animals). The limitations of this paper are that some vitamins are intensively studied and at the same time regarding others, there is a lack of information, especially in animals. Therefore, some subsections are longer and more heavily debated than others. Also, we would like this review to build the foundations for a complex guide regarding the diet for humans and animals with cancer. However, in this case, it is necessary to first focus on the typical nutritional requirements of the species. This means that the information collected in the article below on the positive or negative contribution of individual nutrients to anticancer therapy in mammals should always be tailored to their species’ requirements.

With particular attention to the most common neoplasia, we identified the key scientific words by analyzing several studies published over the last two decades regarding the association between diet and cancer incidence. We excluded studies that, despite being methodologically sound, did not report significant relationships. For each included study, we highlighted the main benefits of the diet in terms of reducing cellular mutation and slowing the progression and spread of the identified pathology. In this framework, an extensive search of the literature was carried out beginning with the scientific and governmental data platforms. The search included the following terms: animal cancer, clinical nutrition, recommendations, survival, cachexia, sarcopenia, malnutrition, and nutritional therapy. Selected sources included English-language guidelines, clinical trials, and observational studies. When applicable, this article included references to seminal articles in the field, even if they were published after the search period.

## 2 The absorption of fat-soluble vitamins in cancer

The body uses vitamins for a wide range of purposes. It can distinguish between two types of vitamins: fat-soluble and water-soluble. In the body, fat-soluble vitamins are transported like lipids and are crucial components of cell membranes. In contrast, water-soluble vitamins are typically coenzymes in metabolic processes that involve the transfer of chemical groups and electrons (21). Vitamins A, D, E, and K are presented below.

Adequate dietary intake, bile acid secretion, micelle production, and adequate duodenal pH in the presence of pancreatic lipase are necessary for the absorption of dietary fat and fat-soluble vitamins (22). The dietary fat-soluble vitamins A, D, E, and K are passively diffused over the brush border after being solubilized in mixed micelles. Vitamin deficiencies can also arise from fat malabsorption due to insufficient bile salts (e.g., bile duct obstruction), lymphangiectasia, or severe villus atrophy. Since vitamin K has small body stores and can cause vitamin K-dependent coagulation factor deficits, especially in cats, this is most significant therapeutically for this vitamin. Retinol, or vitamin A, is consumed either as an ester that needs to be metabolized by pancreatic esterases or as a dimer (beta-carotene) (23). Beta-carotene is taken up immediately from micelles, whereas retinol is insoluble and needs to be attached by a binding protein before absorption. Mixed micelles are the source of vitamin D absorption. It is crucial for maintaining calcium homeostasis, regulating the intestinal absorption of calcium, and modulating renal excretion. Passive diffusion is the method by which vitamin E (α-tocopherol) permeates the lymphatic system from mixed micelles. The intestinal flora synthesizes vitamin K2, while food sources produce vitamin K1. The ileum and colon are likely locations where vitamin K2 is absorbed. Prolonged use of antibiotics can cause a shortage in vitamin K in addition to a bile salt deficit (23).

## 3 Immunomodulatory effects of fat-soluble vitamins in cancer

### 3.1 Vitamin A

It is essential to understand that there are three different forms of vitamin A: retinol, retinoic acid (RA), and retinal. Through the function of nuclear retinoic acid receptors, all-trans retinoic acid, 9-cis retinoic acid, or other metabolites, vitamin A plays an essential role in controlling innate and cell-mediated immunity and antibody response (24). Vitamin A has been found to promote Th1 and Th2 cell growth and differentiation. Consequently, through the inhibition of IL-12 and IFNγ, which are produced by Th1 cells, vitamin A may activate the Th2 anti-inflammatory response (25) (**Table 1**). Likewise, there is data in specific studies that indicate vitamin A correlates significantly with both mitogen-induced pro-inflammatory cytokine (IFN-γ) and anti-inflammatory cytokine (IL-10) (26).

**Table 1:** Fat-soluble vitamin immunomodulatory effects in animal cancer.

Nutrients/category	Anti-inflammatory effects	Pro-inflammatory effects	Refrences
vitamin A vitamin D vitamin E	1. via the inhibition of IL-12 and IFNγ, which are produced by Th1 cells, vitamin A may activate the Th2 anti-inflammatory response 1.Fed diets adequate in calcium and vitamin D, rats fed diets insufficient in these nutrients saw a marked increase in the incidence of breast cancers after being exposed to the carcinogen 7,12-dimethylbenzanthracene (DMBA). It has been shown that calcitriol/VDR in cancer cells enhances the activity of Transforming Growth Factor beta (TGF-β), inhibits mitogenic growth factors (EGF, IGF-1), and activates cyclin-dependent kinase inhibitors, all of which prevent cancerous cells from proliferating. 1. α-tocopherol is the most often utilized and effective form of these, and it is recognized as a powerful antioxidant that neutralizes free radicals and shields biological components from oxidative damage and lipid peroxyl radicals. 2. Vitamin E affects the formation of nitric oxide (NO) via inhibiting transcription nuclear factor kappa B (NF-κB) activation, which is connected to the nitric oxide synthase gene. 3. In dogs, VE resulted in the suppression of monocyte-endothelial cell adhesion, a crucial step in the atherogenic process, and a decrease in IL-1β, a cytokine that can lead to atherosclerosis.	1. RBP4 and NF-κB could function in tandem to enhance the tumor's ability to propagate and metastasize. 2. In the tumor tissue of 67NR/RBP4, but not in the cell culture of these cells, RBP4 overexpression resulted in an increase in VEGF.	(22, 27) (26, 27) (32) (39) (41) (46) (47)
Vitamin K	1.By preventing the release of cytokines like IL6 and the activity of nuclear factor kappa B (NF-κB), vitamin K has an anti-inflammatory impact.	1. Based on genomic databases, vitamin K has been shown to have oncogenic effects in breast and pancreatic cancer by increasing the production of γ-carboxylated proteins. In this way the administration is contraindicated.	(51, 64)

VDR, Vitamin D Recptors; IL-12, Interleukin-1; Th1, T helper cells 1; Th2, T helper cells 2; TNF-κB, Tumor Necrosis Factor-kappa-Beta; NF-κB, Nuclear Factor-Kappa-Beta; ROS, Reactive Oxygen Species; RBP4, Retinol-binding protein 4; VEGF, vascular endothelial growth factor; EGF, Epidermal growth factor, IGF-I, Insulin-like growth factor I; 67NR, nonmetastatic 67NR mammary gland cancer; IL-1β, Interleukin-1 beta; IL-6, Interleukin-6.

Retinol-binding protein 4 (RBP4), vitamin A, and vitamin E are the main subjects of modern obesity research. It has been suggested that RBP4 is an adipokine that connects fat and cancer. Abdominal fat, liver, tumor tissue, and plasma all showed elevated RBP4 levels. Through its direct influence on cancer cells, increased endothelial dysfunction, and impairment of blood arteries within the tumor, RBP4 increases the metastatic potential of breast cancer tumors (27). Few data observed how body weight (BW) growth affects fat-soluble vitamins and related parameters in horses. For 20 months, an excessive energy diet was fed to ten adult gelding Shetland ponies and nine adult Warmblood horses, all of which were healthy and non-obese. The goal was to increase BW. Without changing for ponies and horses, the retinol/RBP4 ratio elevated with BW gain. The increase in the retinol/RBP4 ratio was surprising and requires more explanation when compared to human studies (28).

RBP4 can induce NF-κB, and TNF-α is known to enhance invasion and metastasis by triggering NF-κB signaling, with its expression being higher in 4T1 tumors than in 67NR tumors (29, 30) (**Table 1**). In addition, a study indicates that RBP4 and NF-κB could function in tandem to enhance the tumor’s ability to propagate and metastasize. The finding that NF-κB controls the expression of vascular endothelial growth factor (VEGF) thus contributing to tumor angiogenesis can give support to the possible involvement of NF-κB signaling in the mechanism of the effects of RBP4. Increased expression of RBP4 led to elevated levels of VEGF in tumor tissue from 67NR/RBP4, but this effect was not observed in the cell culture of these cells (31). In the following, vitamin D will be presented.

### 3.2 Vitamin D

Once vitamin D connects to its receptors (VDR), a complex of vitamin D-VDR is formed. This complex can help build a heterodimer compound with the nuclear retinoid X receptor (RXR) or a homodimer with another VDR. Furthermore, the nuclear function can be observed once heterodimers with steroid hormone receptors are formed (32). When vitamin D is attached to VDR or RXR, it can pass through the nuclear membrane, attach to a response element, and stimulate the production of its responsive genes to start a particular gene regulation action (33). A significant quantity of calcium and vitamin D are present in dairy products, and laboratory data suggests that they could decrease the development of breast cancer. On the other hand, conflicting findings resulted from epidemiologic research on dairy products and breast cancer. Nonetheless, both human and animal studies have provided supporting evidence for this theory (34). Vitamin D has been associated with preventing breast cancer in several animal studies. According to research by Jacobson et al. (35), rats given diets deficient in calcium and vitamin D suffered a significant rise in the occurrence of breast tumors following exposure to the carcinogen 7,12-dimethylbenzanthracene (DMBA) compared to rats provided diets with enough calcium and vitamin D (**Table 1**). In chemical carcinogenesis models of breast cancer, it has also been demonstrated that synthetic vitamin D analogs increase tumor latency and decrease tumor incidence and recurrence (36). Additionally, tamoxifen’s ability to stop mammary tumors was significantly increased by the vitamin D analog 1,25-dihydroxy-16-ene-23-yne-26,27-hexafluorochole-calciferol, demonstrating that vitamin D and antiestrogenic compounds may protect against breast cancer through different pathways (37).

Unlike humans, sheep, cattle, horses, pigs, rats, and other mammals, dogs and cats cannot produce vitamin D in their skin by sunlight (38). These species are very dependent on vitamin D from food, so special attention must be paid to this aspect. Given that dogs are carnivores and cats are strict carnivores, this can be explained by the evolutionary adaption of these animals to consume other creatures as prey. If the liver is regularly supplied with vitamin D, these prey animals typically do not suffer from insufficiency because the liver stores vitamin D. Therefore, dogs and cats can rely on their vitamin D reserves if they do not consume prey for an extended period. Since vitamin D is a fat-soluble vitamin, the body can store it (39).

The body responds to calcitriol in a pleiotropic way *via* the VDR receptor, a transcription factor that is a part of the nuclear hormone receptor family. Compared to 25(OH)D, this metabolite can bind to it far more readily (40). Since it was shown that VDR was expressed by a variety of cell types from human and canine tissues, including malignant cells, the non-skeletal roles of vitamin D have been extensively investigated (41). It has been demonstrated that calcitriol/VDR in cancer cells activates cyclin-dependent kinase inhibitors, inhibits mitogenic growth factors (Epidermal growth factor (EGF) EGF, Insulin-like growth factor I (IGF-1), and increases transforming growth factor beta (TGF-β) activity, which stops cell proliferation and the growth of cancer (**Table 1**). It is expressed in the majority of cancerous tissues. Additionally, pro-apoptotic proteins can be activated and anti-apoptotic proteins can be suppressed by calcitriol/VDR signaling, which may also reduce tumor-associated inflammation by suppressing the cyclooxygenase-2, prostaglandin, and NF-kB pathways. Consequently, all previous mechanisms cooperate to suppress the growth of tumors (42).

### 3.3 Vitamin E

Another fat-soluble vitamin with strong antioxidant and antitumoral effects is vitamin E. Eight specific molecules, namely α-, β-, γ-, and δ-tocopherols and their corresponding four tocotrienols, are commonly referred to as vitamins E (43). The most widely used and efficient form of these is α-tocopherol, which is acknowledged as a significant antioxidant that destroys free radicals and protects biological components from harmful oxidative alterations and lipid peroxyl radicals (44). Vitamin E inhibits the amplification of free radical reactions as an antioxidant that breaks down chains (45). As an inhibitor of peroxyl radicals, the vitamin particularly protects polyunsaturated fatty acids found in human plasma lipoproteins and membrane phospholipids (46). Vitamin E levels in plasma influence tissues. Sebum contains a large amount of vitamin E, which is constantly released into the skin’s outer layers (47). Due to oxidative stress’s role in the development of cancer, α-T’s ability to prevent cancer has been thoroughly researched. A higher cancer risk has been associated with reduced VE intake or nutritional status. Certain intervention studies have shown that treating with α-T has a favorable effect on minimizing cancer risk when VE deficiency is present. The focus of more recent research has been on tocotrienols and tocopherols in their γ- and δ-forms (T3). These variants have a significantly lower systemic bioavailability than α-T, yet numerous studies using animal models and cell lines have demonstrated better cancer-preventive actions. In general, γ-T3 and δ-T3 showed greater activity than γ-T and δ-T (48). By blocking the activation of transcription nuclear factor kappa B (NF-κB), which is linked to the production of the nitric oxide NO synthase gene, vitamin E influences the generation of nitric oxide (NO) (49) (**Table 1**). Numerous studies have demonstrated the anti-inflammatory properties of VE, which act on severalenzymes connected to inflammatory reactions, including protein phosphatase and protein kinase C.

Furthermore, treatment with VE in dogs caused the reduction of IL-1β, a cytokine that has the potential to cause atherosclerosis, as well as a suppression of monocyte-endothelial cell adhesion, an essential component of the atherogenic process (50). Low-density lipoprotein oxidation is lowered and decreased coronary heart disease risk has been linked to VE use (51, 52). A fat-soluble vitamin known for its role in coagulation will be presented in the next paragraph. This is vitamin K.

### 3.4 Vitamin K

Natural vitamin K exists in two forms: vitamin K1 and vitamin K2. While vitamin K2 is used to activate numerous extra-hepatic proteins that are more dependent on vitamin K2 than K1, the other is more specific for hepatic activating blood clotting factors. Vitamin K has been demonstrated to have positive effects on the immune system in various diseases, particularly cancer and inflammatory diseases (53). The anti-inflammatory effect of vitamin K occurs by inhibiting the release of cytokines, such as IL6, and the nuclear factor kappa B (NF-κB) activity (54) (**Table 1**).

Most studies on vitamin K have used animals, especially rodents, to investigate its physiology and pathology. Though vitamin K plays the same role in animals as it does in humans, there are some fields, like animal nutrition, where more knowledge of what animals need generally and as they age, may be helpful in their health and well-being (55). In addition to its well-known physiological role, vitamin K has been shown *in vitro* experiments to suppress the proliferation of multiple cancer cell lines (56). While menadione (vitamin K3) is a created version of vitamin K, phylloquinone (vitamin K1) and menaquinones (vitamin K2, MK-n) are found naturally in food (57). Vitamin K has been demonstrated to have anticarcinogenic effects in a number of cell investigations (58–62) as well as a few *in vivo* studies (63, 64). K3, which has potent growth inhibitory effects *via* oxidative stress in many kinds of cancer cell lines, was used in the majority of these experimental investigations (60, 65). Proto-oncogenes like c-myc or c-fos, which promote cell cycle arrest and death, can induce anticancer effects (63). It has been proven that K2 exhibits anticarcinogenic properties in a variety of cancer cell lines, such as those from the breast, colorectum, liver, and stomach (58, 66).

The findings revealed inverse relationships between the total incidence and mortality of cancer and the dietary intake of menaquinones. Genomic databases indicate that in certain types of cancer (pancreatic and breast), vitamin K can exert oncogenic actions *via* the increase of γ-carboxylated protein synthesis (67) (**Table 1**). Therefore, in these types of cancer vitamin K may be contraindicated. Under stressful circumstances, the consumption of dietary supplements containing vitamins E, C, and β-carotene may help prevent imbalances by preventing the generation of free radicals, which in effect increases the normal damage associated with oxidative stress (68).

Vitamin K in the aberrant form known as PIVKA-II is elevated in certain neoplastic illnesses and human coagulation dysfunctions. PIVKA-II concentrations in plasma and tissues can be helpful in veterinary medicine to identify patients with coagulative diseases, but its potential as a marker for hepatocellular carcinoma has not been studied. PIVKA-II acts as a retroactive test to evaluate vitamin K levels (69). This feature has been extensively researched in human medicine, leading to the discovery that elevated PIVKA-II serum concentrations are suggestive of specific neoplastic illnesses, including HCC, and coagulative pathologies (70). In contrast to what has been seen in human HCC, some studies reveal the PIVKA-II unhelpful as a diagnostic or prognostic marker in canine HCC, according to the Maniscalco et al. (71), study, which is restricted to the cohort of cases that were examined (**Table 1**).

## 4 The absorption of water-soluble vitamins

In different species, water-soluble vitamins B and C are absorbed through a combination of active and facilitated transport (e.g., thiamine [B1]), saturable facilitated transport (e.g., riboflavin [B2]), and passive diffusion (e.g., pyridoxine [B6] and C). However, the mechanisms in cats and dogs remain unknown. The more intricate and significant clinical mechanisms of folic acid and vitamin B12 absorption may aid in identifying the kind and location of intestinal illness. Although most commercial foods include sufficient amounts of folic acid, the intestinal flora also produces it. The absorption of folate, also known as pteroyl mono glutamate, occurs through passive diffusion at high amounts and through a carrier-mediated mechanism at low luminal concentrations. The methylation of folate in the cell produces methyl tetrahydrofolate after absorption. In the ileum, vitamin B12, or cobalamin, is absorbed through receptor-mediated endocytosis. However, this process is intricate to separate potentially hazardous analogs from intact cobalamin. R proteins (haptocorrins), nonspecific binding proteins of salivary and gastric origin, attach cobalamin after it is released from food in the stomach. Cobalamin has a strong affinity for R proteins at acidic pH levels. In contrast R proteins bind cobalamin less avidly and undergo proteolysis when they come into exposure to the more alkaline environment of the small intestine (SI) (72). In the ileum, cobalamin is thus transported to the intrinsic factor, another binding protein, which facilitates cobalamin absorption. In dogs, the stomach and pancreas, and in cats, only the pancreas, are the source of intrinsic factor. After passing through the ileum, cobalamin complexes associated with intrinsic factors attach to certain receptors and are endocytosed. The protein transcobalamin 2 binds to cobalamin, allowing it to enter tissues and be reexpelled in bile once it has entered the portal circulation (73).

## 5 Immunomodulatory effects of water-soluble vitamins in cancer

A class of chemical compounds known as water-soluble vitamins are needed by bodies in low quantities to avoid health problems. In recent years, researchers have gained significant insights into the physiological, metabolic, and nutritional functions of water-soluble vitamins. Inadequate effects on a health state can be the result of specific vitamin deficiencies. In most cases, they are usually caused by a poor diet (74).

### 5.1 Vitamin C

The first vitamin presented is C vitamin. Ascorbic acid, also known as vitamin C, is an organic substance formed from glucose molecules known as lactone, which is a gluonic acid. In the bloodstream, it is the most prevalent water-soluble antioxidant. Leukocytes, salivary glands, the pancreas, kidney, small intestine, brain, lymph nodes, lungs, testicles, and spleen are just a few of the tissues and organs that contain it (75). The two major biological forms of vitamin C are its oxidized form (dehydroascorbic acid) and its reduced form (ascorbic acid). Due to its general qualities or its function as a pro-oxidant in high concentration, vitamin C has been connected with the prevention, progression, and treatment of cancer (76).

Certain transporters, like sodium-dependent vitamin C transporters, and glucose transporters are responsible for transporting the reduced form into the cells. Glutathione oxidizes it to dehydroascorbic acid, which is then reduced back to ascorbic acid. As an antioxidant, vitamin C combats free radicals and contributes to the production of carnitine as well as type I and type II collagen (77). The livers of the majority of mammals naturally synthesize vitamin C from glucose and galactose. Acid reduction is critical to the health of mitochondria, which require antioxidant systems to neutralize oxidative phosphorylation, as mitochondrial DNA is more susceptible to oxidative damage than nucleus DNA. Under oxidative stress, vitamin C gives protection (77) (**Table 2**).

**Table 2:** Water-soluble vitamin immunomodulatory effects in animal cancer.

Nutrients/category	Anti-inflammatory effects	Pro-inflammatory effects	Refrences
vitamin C vitamin B1 vitamin B2 Vitamin B3	1. Vitamin C offers protection against oxidative stress. 2. Although these effects have been demonstrated to decrease the synthesis of antioxidant defense, impair proteolytic activity, and stimulate the buildup of oxidized proteins, vitamin C can offset the effects of long-term modifications that can generate free radicals. 1. Cancer patients had 20% lower blood levels of thiamine and thiamine diphosphate (TDP), 20% lower transketolase activity, and a 5-to 42% lower TDP impact. 1.Riboflavin deficiency has been associated with inhibiting tumor growth in both animal models and cell studies. 2. A few cohort studies and meta-analyses have linked total riboflavin intake from meals and supplements to a lower risk of colon cancer. 1.There is a positive correlation between the mRNA levels of genes associated to NAD+ production and the expression of genes influencing muscle mitochondrial biogenesis, muscle mass growth, and muscle regeneration in animals. 2. Niacin in dogs (100 mg/day) has the potential to reduce hypertriglyceridemia by inhibiting the release of fatty acids from adipocytes and the formation of VLDL.	1.Thiamine supplementation may be the reason of the high rate of tumor cell proliferation, survival, and resistance to chemotherapy. 2. Furthermore, thiamine has been connected to cancer through its effects on reactive oxygen species, cyclooxygenase-2, prostaglandins, matrix metalloproteinases, and nitric oxide synthase. 1. High-dose of RF supplementation was important in inducing cancer cells to proliferate, invade, and migrate. 1. Erythema, pruritus, abnormal liver function test results, vomiting, and diarrhea are potential niacin side effects.	(71) (80, 86) (80) (88, 90) (91, 92) (105, 109) (107, 109)
Vitamin B5 Vitamin B6 Vitamin B7 Vitamin B9 Vitamin B10 (PABA) Vitamin B12 Vitamin B13 (Orotate) Vitamin B17 (Amygdalin)	1.Vitamin B5 and CoA promote the differentiation of CD8 + cytotoxic T cells into Tc22 cells that generate interleukin-22 (IL-22). 1. The potential of vitamin B6 to prevent DNA damage may contribute to its anticancer properties. 1.The absence of biotin affects the expression of transcription factors like NF-κB and SP1/3, indicating that biotin regulates immunological phenomena via mechanisms besides carboxylation and decarboxylation 1.In primary human lymphocytes, folate deprivation decreased cell proliferation and increased apoptosis, cell cycle arrest, and DNA strand damage. 1. PABA could be an agent that increases the anticancer activity of ionizing radiation through a process that involves changes in the expression of proteins that control cell cycle arrest. 1. Deficiency in vitamin B12 leads to a higher rate of uracil misincorporation, which impedes DNA synthesis and culminates in genomic instability. 2. After receiving NO-Cbl treatment for ten weeks, the Giant Schnauzer's tumor volume had decreased by 77%. 3. A promising anti-cancer treatment that capitalizes on the tumor-specific properties of the vitamin B12 receptor is the use of NO-Cbl in dogs. 1. Magnesium orotate, a combination of magnesium and orotic acid, can be used as an adjuvant treatment for type 2 diabetes, hypertension, congestive heart failure, and post-operative cardiac condition. 1 Thus, amygdalin has been shown in vitro experiments to induce apoptosis due to upregulated expression of caspase-3 and Bax protein and downregulated expression of Bcl-2, an anti-apoptotic protein.	1. To sustain one-carbon metabolism and the growth of tumor cells, pancreatic ductal adenocarcinoma (PDAC) cells actively consume VB6, depriving the tumor microenvironment of VB6.	(110) (124, 127) (140) (153) (169) (181) (182) (182) (185, 186) (205)

TDP, thiamine diphosphate; NAD^+^, Nicotinamide adenine dinucleotide; VLDL, Very Low-Density Lipoprotein; NO-Cbl, nitrosylcobalamin.

In summary, research on the connection between vitamin C and cancer is ongoing. Differences in results are due to a combination of factors including ascorbic acid-related concerns (doses, administration routes, plasmatic levels, metabolites recording, and source), cancer characteristics (specific mutations present, type and grade of cancer, received conventional anti-cancer therapy), and individual characteristics (diet, behaviors, genetics, transporters). This connects the recording of doses to an active anti-cancer mechanism and makes them effective (76).

Vitamin C improved older horses’ antibody response to vaccinations, particularly in animals with Cushing’s syndrome or pituitary dysfunction. Encourage recovery and eliminate blockages in the airways. Vitamin C can counteract the consequences of long-term alterations that can generate free radicals, even though these effects have been shown to inhibit the production of antioxidant defense, lower proteolytic activity, and encourage the buildup of oxidized proteins (78, 79). The next paragraph underlines the essential roles in the immunomodulation of B vitamins.

It has recently been demonstrated that a variety of B vitamins, all of which are generated by the microbiota to some extent, are essential for the immuno-regulatory activity of the gut microflora.

For instance, nicotinic acid, or vitamin B3, sometimes referred to as niacin, is known to have anti-inflammatory properties that help prevent colon cancer in rats (80). Clinical experiments using nicotinamide (NAM), a derivative of vitamin B3, provided evidence in favor of the hypothesis that vitamin B3 mediates efficient chemoprevention against non-melanoma skin cancer (81). Recent research analyzed the methods by which NAM may prevent the development and start of luminal B breast cancer in mice, demonstrating that NAM activates T and NK cells involved in immuno-surveillance (82). Notably, gemcitabine and anthracycline-based immunogenic chemotherapy are particularly beneficial when used in conjunction with NAM in preclinical models of pancreatic and breast carcinoma (83).

### 5.2 Vitamin B1

The first vitamin presented is Vitamin B1 (Thiamine). In mammals, thiamine, often known as vitamin B1, is a water-soluble vitamin that is crucial for a healthy diet. As a nutrient that is frequently deficient in ruminants, thiamine has received a lot of attention. This is mainly because the rumen bacteria inactivate the vitamin, causing distinctive cerebrocortical necrosis and neurologic symptoms (84). It plays a vital role as a cofactor in the metabolism of carbohydrates, the synthesis of nucleotides and nicotinamide adenine dinucleotide (NAD), and the nervous system when it takes the form of thiamine diphosphate (TDP). Thiamine is involved in a number of important biochemical processes that occur in the body, including the pentose phosphate pathway and the TCA cycle, which are processes involved in the metabolism of carbohydrates. Additional forms of thiamine contain thiamine that has one or three phosphate groups (thiamine monophosphate and triphosphate, respectively) and remains unphosphorylated (free). Thiamine works as a co-enzyme for complexes including enzymes essential for intracellular glucose metabolism, such as transketolase, pyruvate dehydrogenase (PDH), and α-ketoglutarate dehydrogenase. Moreover, thiamine controls the expression of genes that produce the cofactor thiamine-using enzymes. Transketolase, PDH, and mRNA levels are decreased by thiamine deprivation (85). Thiamine and thiamine diphosphate (TDP) concentrations are reduced by 20% in cancer patients’ blood, transketolase activity is reduced by 20%, and the TDP impact is reduced by 5–42% (86) (**Table 2**).

Since dogs and cats cannot synthesize thiamine, they must consume thiamine through food. As per the dietary needs established by the National Research Council (NRC), cats need between two and four times the daily amount of thiamine compared to dogs (27, 87).

There is debate regarding thiamine’s involvement in cancer. The high rate of tumor cell survival, growth, and resistance to chemotherapy may be caused by thiamine supplementation (88) (**Table 2**). Additionally, thiamine’s effects on prostaglandins, reactive oxygen species, cyclooxygenase-2, matrix metalloproteinases, and nitric oxide synthase have been linked to cancer (89). Nevertheless, some research has indicated that thiamine might have some anticancer properties. In a case-control study, higher intakes of vitamin C, beta-carotene, thiamine, and nicotinic acid were also linked to a lower risk of stomach cancer (90). Moreover, leukocytes and blood plasma from acute leukemia patients showed reduced thiamine levels (91). Patients with breast and bronchial carcinomas had significant levels of thiamine excretion in their urine and the thiamine pyrophosphate (TPP) stimulating action, indicating a possible thiamine deficit in these individuals (92).

### 5.3 Vitamin B2

As expected in this paragraph Vitamin B2 is presented. Also, known as riboflavin (RF), is an essential component of two primary coenzymes: flavin mononucleotide (FMN) and flavin adenine dinucleotide (FAD). These coenzymes are crucial components of intracellular biochemistry and play key roles in energy production, cellular function, development, and metabolism (93). Remarkably, in both animal models and cell investigations, RF deprivation has been linked to the prevention of tumor growth (94) (**Table 2**). Research revealed that high-dose riboflavin supplementation was important in inducing cancer cells to proliferate, invade, and migrate (95, 96). There is insufficient data associating riboflavin with the prevention or cancer treatment, and research results are ambiguous (95, 97). Also, findings from observational studies on the relationship between riboflavin consumption and the risk of CRC were contradictory. Total riboflavin intake from foods and supplements has been associated with a decreased risk of colorectal cancer, according to a few cohort studies and meta-analyses (97, 98) (**Table 2**). Additionally, there are inconclusive results from case-control studies (99–101), as well as individual cohort studies (102, 103). Regarding CRC in different patients, a directly proportional link between the serum RF amount and the risk of malignancy was found. After controlling for several variables, including sex, age, history of polyps, medical conditions, medications, BMI, and another CRC-related nutritional status, the association between riboflavin and CRC risk remained and showed a dose-response link (104).

In a dose-dependent manner, vitamins B2, B6 (pyridoxine), and B9 (folic acid) inhibit the proliferation and migration of U937 cells. Only a smaller number of cells develop under standard culture conditions when either vitamin B2, B6, or B9 is present. The cells generated in these settings are healthy and alive. The synthesis of energy, antioxidant defense, and homocysteine metabolism are all correlated with vitamin B2. A lack of vitamin B2 is linked to anemia, neurotoxicity, growth retardation, and potentially some types of cancer (105). Indeed, it has recently been demonstrated that riboflavin lack increases cell proliferation and decreases cell viability in HepG2 cells (106).

In addition, during pregnancy and lactation, the mammary gland substantially induces the multidrug transporter breast cancer resistance protein (BCRP/ABCG2). It has been shown that riboflavin, or vitamin B2, is pumped into milk by BCRP, providing this vital ingredient to the development. Riboflavin was secreted in milk at a rate that was >60 times lower in Bcrp1−/− mice than in wild-type mice. Nonetheless, in experiments, Bcrp1−/− pups did not exhibit riboflavin shortage because of concurrent milk secretion of flavin adenine dinucleotide, which is its cofactor (107).

In animals, pigs with RF deficiencies have decreased growth performance and less appetite. In extreme circumstances, it may result in piglets’ mortality and cause the swine farm to suffer significant financial consequences (108). Since most plant diets are deficient in RF, piglets’ meals usually require to be supplemented with RF (108).

Diet composition changes play an important part in the maturation of gastrointestinal function during weaning. Currently, dietary modifications have been linked to both qualitative and quantitative changes in the gastrointestinal system in animal studies. When the riboflavin lack was corrected, some of the early morphologic and cell motility alterations in the gastrointestinal tract were observed in weanling rats given a riboflavin-deficient diet until weaning was irreversible (109, 110). As is expected, the next vitamin with an essential role in cancer metabolism is Niacin.

### 5.4 Vitamin B3

Niacin, Vitamin B3, water-soluble and resistant to light and oxidation, is a vitamin that may be found in both acidic and alkaline environments. The precursor of nicotinamide adenine dinucleotide phosphate (NADP) and nicotinamide adenine dinucleotide (NAD) is niacin or vitamin B3. In addition to being essential for energy metabolism, good digestion, and the preservation of a healthy neurological system, niacin also supports good skin. Changes in NAD^+^ activities can impact tissue function since it is a necessary cofactor for several mitochondrial redox processes and can therefore interfere with mitochondrial homeostasis. The expression of genes controlling muscle mitochondrial biogenesis, muscle mass growth, and muscle regeneration in mice is favorably correlated with the mRNA levels of NAD^+^ biosynthesis-related genes giving validity to this theory. Sarcopenia and mitochondrial myopathy are two examples of muscle illnesses for which NAD^+^ has been identified in recent animal and human research as a pathological characteristic (111) (**Table 2**). Niacin and tryptophan metabolism are closely associated. If dietary tryptophan is sufficient and niacin levels are low, niacin can also be produced from tryptophan.

Animals, particularly cats cannot convert the amino acid tryptophan into niacin, whereas dogs can. For this reason, cats need niacin in their diet more often than dogs do (112). Niacin (100 mg/day in dogs) may also lower hypertriglyceridemia by decreasing the production of VLDL and the release of fatty acids from adipocytes (113, 114) (**Table 2**). Erythema, pruritus, abnormal liver function test results, vomiting, and diarrhea are potential niacin side effects. The use of niacin in dogs and cats is limited due to these side effects and the absence of strong evidence of benefit. Horses fed a diet high in protein could require more niacin. *Via* mitochondrial malfunction and ROS activation caused by bifurcating metabolic pathways (reverse electron transport and lipid metabolism), nicotinamide therapy promotes cancer cell death in TNBC in humans and mice (115) (**Table 2**).

### 5.5 Vitamin B5

In this subsection it is discussed about the vitamin B5 (Pantothenic acid).The majority of foods include pantothenic acid, or vitamin B5, which is created by the gut bacteria and is a precursor to coenzyme A (CoA). According to a recent study in mice, vitamin B5 and CoA promote the differentiation of CD8 + cytotoxic T cells into Tc22 cells that generate interleukin-22 (IL-22), most likely by enhancing mitochondrial metabolism (116) (**Table 2**). More frequently acyl-CoA derivatives, which act as “activated” fatty acids to take part in intracellular fatty acid transport and lipid biosynthesis, and CoA can be conjugated to acetate to form acetyl-CoA thioester, which is crucial in the connection of amino acid catabolism, glycolysis, and fatty acid metabolism (117, 118). It has recently been demonstrated that a variety of B vitamins, all of which are generated by the microbiota to some extent, are essential for the immune-regulatory activity of the gut microflora.

First of all, elevated amounts of vitamin B5 may simply be the result of a balanced diet and microbiota, necessary for a general state of health or “fitness” that encourages effective defenses against infections or cancerous cells (119). Additionally, the dietary fiber component inulin is effectively converted into vitamin B5 by the fecal microbiota (120), and dietary fiber abundance improves the response to immunotherapy in patients with melanoma (121).

A family of proteins expressed by vanin genes has provided more information on the function of pantothenic acid. CoA catabolism produces pantetheine, which vanins, also called pantetheinases, work with to produce pantothenate and cysteamine, the latter of which increases inflammation (122). Mice without vanin-1 showed tolerance for apoptotic oxidative tissue damage due to paraquot or γ-irradiation (123). Additionally, these mice diminished chemically generated inflammation in colitis models (124). Vanin-1 activity is thought to counteract PPARγ, as evidenced by the anti-inflammatory phenotypes of vanin-1-deficient mice that PPARγ inhibitors inhibited (124).

In the context of cancer immunotherapy, a relative investigation supported the notion of vitamin B5-mediated immunostimulatory effects. The authors initially assessed the metabolic characteristics pathways of different effector CD8+ T cell subpopulations, that can be identified based on their cytokine profile. Thus, they can be Tc1 (producing interferon-γ and interleukin [IL]-2), Tc17 (producing IL-17), and Tc22 (producing IL-2 and IL-22), in order to characterize the role of antitumor T cells in immunotherapy (125). The development of Tc22 cells, which are highly effective anticancer effects, requires a metabolic remodeling process toward oxidative phosphorylation and, consequently, mitochondrial ATP synthesis. Using mouse Tc1, Tc17, and Tc22 T cells that had received *in vitro* differentiation, mass spectrometric metabolomic investigations were conducted to identify the metabolic causes of Tc22 polarization.

These investigations showed that Tc22 cells have high vitamin B5 and CoA levels (125). Additionally, the *in vitro* differentiation of Tc22 in the presence of exogenous CoA led to increased oxidative phosphorylation, the production of reactive oxygen species (ROS) by the mitochondria, higher levels of cellular ATP, and an increase in the production of IL-2 and IL-22. This was achieved through the incorporation of glucose-derived 13C into tricyclic acid cycle (TCA) metabolites (126). The activation of two transcription factors, aryl hydrocarbon receptor (AhR, which is sensitive to ROS) and hypoxia-inducible factor (HIF)-1α, which is responsive to the TCA metabolites succinate, were associated with an increase in IL-22 production. Crucially, tumor antigen-specific T lymphocytes demonstrated improved tumor growth-reducing capacities when stimulated *in vitro* with CoA and subsequently injected into transgenic mice expressing this antigen in pancreatic islet tumors (125).

Compared to a healthy control group, dogs with cancer exhibited lower levels of biotin and 25-hydroxycholecalciferol, but higher levels of retinyl palmitate, ascorbic acid, thiamine pyrophosphate, and flavin mononucleotide. There was no difference in the levels of retinol, retinyl stearate, alpha-tocopherol, riboflavin, flavin, adenine dinucleotide, pyridoxal-5′-phosphate, cobalamin, folate, and pantothenate between dogs in good condition and those in sickness (127). The next subsection presents the immunomodulatory effects of Pyridoxine.

### 5.6 Vitamin B6 (Pyridoxine)

Pyridoxine hydrochloride and pyridoxal-5-phosphate an essential components for hormones, proteins, and neurotransmitters—chemicals that transmit messages between nerve cells. In the body’s cells, vitamin B6 functions as a coenzyme in more pathways, including transamination, which is the process of adding nitrogen to a fatty acid to generate an amino acid, and decarboxylation, which is the reaction of removing a carbon atom in to reduce an amino acid chain (128).

According to Komatsu et al. (129), vitamin B6 intake significantly reduced the growth of colon cancer-causing azoxymethane-induced cells in mice. To sustain one-carbon metabolism and the growth of tumor cells, pancreatic ductal adenocarcinoma (PDAC) cells actively consume VB6, depriving the tumor microenvironment of VB6. On the other hand, VB6 is necessary for the intracellular breakdown of glycogen, which is a vital source of energy for the activation of natural killer (NK) cells. When one-carbon metabolism is blocked along with VB6 supplementation, the tumor burden is significantly reduced *in vivo* (130) (**Table 2**). The incidence of CRC was inversely associated with vitamin B6 intake and blood PLP levels (131). According to Ames and Wakimoto (132), PLP has been suggested to impact carcinogenesis *via* a variety of mechanisms, including those involving DNA metabolism. This suggests that vitamin VB6’s anticancer characteristics may partly result from its ability to prevent DNA damage. Moreover, diabetes and VB6 have been related. Whether low PLP levels are a cause, a consequence, or both of diabetes is unclear, though. According to some research, low PLP levels may be a contributing factor in the development of diabetes, whereas other studies demonstrate that diabetes lowers PLP levels. There is growing information that individuals with diabetes are more likely to develop several forms of cancer, and numerous studies have correlated insufficient vitamin B6 intake to an increased risk of cancer (133) (**Table 2**). Due to prevalent risks, both cancer and diabetes are connected. A growing amount of research indicates that individuals with diabetes mellitus are more likely to develop cancer due to a variety of unclear causes, involving impairment to their DNA (134, 135). Hyperglycemia is responsible for cell proliferation and oxidative stress because too much glucose *via* multiple paths stimulates the production of reactive oxygen species (ROS), which damages DNA and other cells (136). Furthermore, decreased protection against antioxidants along with insufficient repair of DNA in diabetes cells increases DNA damage (137). Both type 1 and type 2 diabetes patients are frequently observed to have DNA strand breaks and oxidative damage (138, 139).

Despite the abundance of human studies in this area, very little research exists on the B6 status of household animals, including cats. Because of their high protein needs, cats have a higher requirement for vitamin B6 (pyridoxine).Twelve studies (eight original research publications) covering the period from 1959 to 1998 have examined cats’ vitamin B6 levels. In cats, vitamin B6 deficiency causes microcytic hypochromic anemia with high serum iron, convulsions, kidney lesions, failure to grow, emaciation, convulsions, anemia, oxalate nephrocalcinosis, ataxia, and, if left on the diet, seizures, and death, according to the first three studies conducted in the late 50s and early 60s (140). Five studies covering the years 1989 to 1998 were published, separated by over 28 years. A lack of vitamin B6 in growing kittens resulted in decreased food intake, pyridoxal phosphate, pyridoxal, hemoglobin, and hematocrit in plasma, along with higher levels of renal oxalate, blood tyrosine, and blood cystathionine (141).

Additionally, aberrant histology, specifically active tubular degeneration and oxalate deposition, was observed in growing kittens that were deficient in vitamin B6 (142). Growing kittens’ dietary protein concentrations, like in people, mice, and chickens, affected their B6 requirements. For example, kittens fed a 30% casein diet needed 1-2 mg of pyridoxine/kg food, but those fed a 60% casein diet needed > 2 mg (143). The discovery that vitamin B6 deficiency in cats impacted brainstem auditory evoked potentials. It connected extended inter-wave intervals to decreased axonal conduction velocity due to faulty myelination offered a fresh extension of previous observations (144).

In a study, 41 dogs with non-Hodgkin’s lymphoma were randomly assigned to receive oral pyridoxine or a placebo dailywhile receiving Doxil chemotherapy in a double-blind method. Although pyridoxine did not erase palmar-plantar erythrodysesthesia (PPES), it did so later and less drastically than in dogs given a placebo. This led to fewer treatment delays or changes, which allowed for a higher cumulative dosage of Doxil to be administered. Dogs treated with pyridoxine obtained a median cumulative dose of 4.7 mg/kg (mean, 4.1 mg/kg) compared to the 5.0 mg/kg cumulative goal dose, while dogs treated with placebo received a median of 2.75 mg/kg (mean, 2.9 mg/kg; P). In this canine model, it was found that pyridoxine is useful in postponing the onset and severity of PPES (145).

### 5.7 Vitamin B7

In this subsection Vitamin B7 (Biotin) is presented.Although biotin can be found in many foods that are included in a typical diet, it usually appears in a form that is bonded to proteins and cannot be readily utilized by cells. The pancreatic enzyme biotinidase releases peptide- or lysine-bound (biotinyl-lysine) biotin, which is then taken up by various transporters and absorbed into cells (146). Once within the cell, biotin becomes covalently attached to the biotin carboxyl carrier protein (BCCP), which functions as a prosthetic group for several carboxylases/decarboxylases that control the production of fatty acids, gluconeogenesis, lipogenesis, and the degradation of valine and isovalerate in addition to other branched-chain amino acids. Since it has been shown that deficiency affects the expression of different genes in the liver, including NO-like actions that raise cGMP through elevated guanylate cyclase, biotin exerts a secondary role in controlling gene expression (147).

In the gut microbiome, bacteria produce biotin *via* two main pathways. In the first, malonyl-acyl carrier protein (ACP) is transformed to malonyl-ACP methyl ester, which is then further converted to pimeloyl-ACP methyl ester. Pimeloyl-ACP methyl ester can also be directly produced as a substrate. A third process converts pimelate into pimeloyl-CoA by using it as a substrate. Biotin is subsequently generated through the combination of these pathways in a four-step process (148).

Increased inflammation is linked to a deficit in biotin (149). Neurodegenerative illnesses are linked to oxidative stress, cell death, and dysfunctional mitochondria. Biotin treatment reduced oxygen free radicals and apoptosis while partially restoring mitochondrial activity in myelin-producing oligodendrocytes (150). When exposed to LPS, monocyte-derived DCs grown in a medium lacking in biotin generated higher levels of inflammatory cytokines (149). BMI was found to be negatively linked with serum biotin levels, inflammation, and hypertriglyceridemia in a study involving monozygotic twins who were discordant for BMI (151). The absence of biotin affects the expression of transcription factors like NF-κB and SP1/3, indicating that biotin regulates immunological phenomena *via* mechanisms besides carboxylation and decarboxylation (152) (**Table 2**). Even though biotin’s molecular targets have been thoroughly investigated, little is now known about how biotin functions in immune modulation and cancer prevention.

Multiple studies demonstrate tumor cells that overexpress biotin-selective transporters as potential beneficial absorbers of biotin or biotin-conjugates. In these conditions, a biotin component is likely an appropriate choice for target delivery, biosensing, and live-cell imaging (153). Biotin has been used as a conjugation for a variety of targeted imaging, sensing, and delivery applications both *in vitro* and *in vivo* because of its strong affinity for BRs. It has been shown that biotin’s affinity for BR is unaffected by chemical changes made to its carboxylic acid group (154).

Since proliferating cancer cells have many receptors for biotin, also known as vitamin B7, vitamin H, and coenzyme R, which are critical for the uptake of vitamins, biotin has been connected to the semi-synthetic analog docetaxel (DTX). It has recently been demonstrated that in many cancer cell lines, including leukemia (L1210FR), ovarian (Ov2008, ID8), colon (Colo-26), mastocytoma (P815), lung (M109), renal (RENCA, RD0995), and breast (4T1, JC, MMT06056) cancer cell lines, biotin receptors are overexpressed more than folate and/or vitamin B-12 receptors (155, 156).

Among the taxanes used to treat various malignancies is DTX (157). Furthermore, in order to achieve anticancer drug delivery, well-established mesoporous silica nanoparticles (MSNs) have been selected as carriers of the named metallodrugs. To enable the selective release of essential drugs inside tumors, chitosan (CTS) coupled with biotin, a pH-sensitive additive, is attached to MSNs (158). Also, biotin is involved in breast cancer therapy. The most common treatment for breast cancer is radiotherapy (RT), yet due to the minimal variation in how normal tissues and tumors react to ionizing radiation, RT has severe adverse effects. In this way, an UiO-66-NH2@AuNS core-shell nanoparticle was created. After that, the solid gold shell was scratched into solid AuNS (HAuNS), and to create HAuNS@PEG-bio, it was further altered using biotin-PEG-SH (PEG-bio). The near-infrared II (NIR-II) area photothermal therapy (PTT) performance of HAuNS@PEG-bio is demonstrated to be effective, and the elevation of temperature at the tumor site stimulates blood circulation to mitigate the hypoxia within the tumor microenvironment (TME) (159).

Chemotherapy and extended antibiotic therapy were administered to a female dog who had a sticker tumor and recurrent cystitis. After a few months of treatment, lesions with hyperkeratosis, skin thickness, bleeding fissures, and inflammation occurred across the nasal area and palmar and plantar regions. During the patient’s 60-day treatment, which included 15 mg of oral biotin supplementation (1.4 mg kg-1 of body weight) once daily, the patient’s skin lesions significantly improved. According to these findings, intestinal biotin production may not be sufficient in some conditions, especially those requiring extended antibiotic therapy, necessitating oral supplementation (160). In the following paragraph, vitamin B9 is presented.

### 5.8 Vitamin B9 (Folate)

Dark-green leafy vegetables and legumes are among the foods that contain folate, an important water-soluble B vitamin. The synthetic version of folic acid is found in fortified foods including cereals and grains, as well as supplements. While folic acid is readily accessible in its oxidized pteroylmonoglutamate form, dietary folate is present in a reduced state with polyglutamate side chains that require oxidation and hydrolysis for absorption (161).

Folate has been thoroughly investigated as a potential pathway for the development of cancer because of its involvement in one-carbon metabolism. In the methionine pathway, folate in the form of 5-methyltetrahydrofolate (5-MTHF) and cobalamin are necessary for the conversion of homocysteine to methionine. S-adenosylmethionine (SAM) is produced from methionine (162). SAM is a major methyl donor to numerous bodily processes, such as the methylation of DNA and RNA (162). Reduced methylation of CpG islands in DNA, which affects gene transcription and modifies the expression of proto-oncogenes and tumor suppressor genes, may result from insufficient SAM synthesis (163). Moreover, low folate levels can hinder the conversion of dUMP (deoxyuridine monophosphate) into dTMP (deoxythymidine monophosphate), which is an essential nucleic acid for DNA synthesis and repair. Mistaken uracil substitution for thymidine can eventually result in strand breakage, unstable DNA, and defective DNA repair (163).

Certain immunological functions have been demonstrated to suffer from folate deprivation. Cell proliferation is promoted when CD8+ T lymphocytes are treated with phytohaemagglutinin and IL-2. However, stimulation was suppressed without folic acid. This activity did not affect CD4+ T cells (164). In primary human lymphocytes, folate deprivation decreased cell proliferation and increased apoptosis, cell cycle arrest, and DNA strand damage (165) (**Table 2**). Some research found that compared to aged corresponding controls, newly diagnosed cancer patients had mean levels of homocysteine significantly higher and vitamin B9 levels significantly lower. This suggests that low folate and high homocysteine may be linked to lung cancer, although more research is needed to confirm these findings (166). High levels of homocysteine could have a consequence on the methylation of specific genes that regulate the initiation and progression of breast cancer, according to a different study on the disease. As a result of increased homocysteine concentrations, the breast cancer cell lines MCF-7 and MDA-MB-231 revealed epigenetic modulations of BRACA1 and RASS-F1 (167). Although elevated levels of the enzyme PDXK, which helps in the conversion of pyridoxine, the precursor to vitamin B6, into pyridoxal-5′-phosphate, the bioactive form of vitamin B6, have also been related to an increased risk of colorectal cancer developed plasma homocysteine levels have been linked as well with an increased risk of non-small cell lung carcinoma (168, 169).

Deficits in B vitamins can raise homocysteine levels, which can then cause DNA damage, oxidative stress, and a persistent inflammatory state that can modify epigenetics and cause cancer (170). Certain B vitamins, such as vitamin B6, have been shown to function as antioxidant nutrients and, as such, to prevent inflammation and the advancement of cancer (171). Circulating blood cells called monocytes can develop into dendritic or macrophage cells at specific tissue locations. Specialized antigen-presenting cells, such as macrophages and dendritic cells, process, present, and release cytokines to T cells (172). A kind of cancer known as histiocytic lymphoma is an aggressive non-Hodgkin’s lymphoma that develops from immune system cells, primarily from monocyte pro-monocytic blast origin.

Divergent results from research on animals suggest that the impact of folate on neoplasia varies depending on the animal and tumor model, the kind, time, amount, and duration of the carcinogen application, the stage of carcinogenesis, and the amount and type of folate given. The relationship between folate and cancer of the cervix, colorectum, lung, esophagus, and brain has been studied epidemiologically. The results indicate that low folate concentration may be a significant factor early in the neoplastic process. More relevant is the possibility of suppressing precursor lesions in the cervix and colorectum, specifically adenomatous polyps and cervical intraepithelial neoplasia. Methotrexate suppresses the function of folate by reducing the intracellular synthesis of tetrahydrofolates from dihydrofolates. It is a chemotherapeutic drug used for the treatment of acute lymphocytic leukemia and choriocarcinoma, among other neoplasms. Shklar and coworkers (173) examined the effects of methotrexate on hamster buccal pouch carcinomas generated by 9,10-dimethyl-l,2-benzanthracene and found that the methotrexate-treated group reported tumors that were larger and more anaplastic and that they progressed more quickly than the control group. In a different study, the impact of methotrexate was evaluated in a Swiss mouse skin tumor model methylcholanthrene (MCA). The incidence of papillomas was 74% in animals fed the control diet and 96% in mice fed a diet supplemented with methotrexate six weeks before, during, and after MCA treatment. Nevertheless, tumor development was limited to 36% of mice that received methotrexate just one week before, during, and following MCA treatment. Hence, methotrexate may have an anticancer or cocarcinogenic impact, depending on when and how long it is used (174).

However, experimental data indicates that a lack of folate could have beneficial effects on cancer while taking folate supplements could accelerate the development or growth of malignancies. For instance, Baggott and colleagues (175) gave rats meals containing varying amounts of folic acid (0, 2, or 40 mg/kg diet) or folinic acid (20 mg/kg diet). Following the start of their diets, rats were administered methylnitrosourea to cause breast cancer and followed by full diets with 2 mg/kg of folic acid immediately. The frequency of tumors did not change substantially among the groups. Nevertheless, the folate-supplemented groups had a greater rate of mammary malignancies per tumor-bearing animal, and these cancers manifested earlier (175). In the next paragraph it will present vitamin B10.

### 5.9 Vitamin B10

The chemical molecule para-aminobenzoic acid (PABA) is also referred to the name vitamin B10 (or vitamin Bx). The nutrient PABA is necessary for many mammal diseases but unnecessary for the human body, and its derivatives have demonstrated various biological actions. The chemical PABA is non-toxic and readily absorbed in the intestine, and its derivatives exhibit a wide range of biological activity (176). It’s thought that PABA scaffold-containing medications are well tolerated (177). PABA is an essential and unique vitamin belonging to group B that is required to synthesize folic acid (178).

According to Maki and Takeda (179), bacteria, yeast, and plants all produce this vitamin. PABA is necessary for the production of folic acid. PABA is well-known for its function in triggering the production of interferon, which has an antiviral impact, in humans (180). While radiation did not affect melanocytes, PABA increased (by 50%) the growth-inhibitory effects of radiation on B16F10 cells. While applied to B16F10 and 4T1 tumors *in vivo*, PABA increased (by 50–80%) the anticancer effect of radiation. PABA and radiation treatment together accelerated the death of tumor cells. After PABA was added to tumor cells, CDC25A expression increased while p21CIP1 levels fell. PABA could be an agent that increases the anticancer activity of ionizing radiation through a process that involves changes in the expression of proteins that control cell cycle arrest (181) (**Table 2**). PABA and its derivatives have been used as an anti-reactive oxygen species as active ingredients in sunscreens to provide UVB protection (182). Additionally, PABA showed strong anticoagulant and beneficial agent effects (180, 183). Diagnostic tests for gastrointestinal tract conditions based on PABA (para-aminobenzoic acid) are now available (184). Skin conditions such as scleroderma, dermatomyositis, and Peyronie’s disease are the primary conditions treated with potassium 4-aminobenzoate (185).

Cancer is analyzed as being the second most prevalent reason for death, behind cardiovascular diseases (186). Because they have fewer side effects and are more selective for malignant cells, targeted chemotherapeutical drugs are preferable to standard ones (187). Conventional anti-folates such as aminopterin, pralatrexate, methotrexate (MTX), and pemetrexed (PMX) are the most prevalent types of DHFR inhibitors. The three major components that make up the chemical structure of the MTX model are the p-amino benzoic acid, glutamic acid, and pteridine nucleus (188). As anti-inflammatory, anti-diabetic, antibacterial, and antioxidant chemicals, certain 1,2,4-triazoloquinazolines have been reported to be beneficial (189).

A particular substrate for pancreatic chymotrypsin, the synthetic peptide N-benzoyl-L-tyrosyl-p-aminobenzoic acid (BT-PABA), has been used to measure exocrine pancreatic insufficiency in dogs through oral administration. The next step was testing for PABA in the plasma or urine, which could distinguish between control animals and those suffering from exocrine pancreatic insufficiency (EPI) without affecting the outcome when the xylose absorption test was combined with pancreatic function. Possible interference with the peptide test’s specificity for diagnosing EPI was investigated in six small intestinal disease-afflicted dogs. These results indicate that modest intestinal anomalies do not significantly impact PABA absorption to compromise the peptide test’s specificity in identifying severe EPI in dogs. On rare occasions, small intestinal illness may be the secondary cause of this deficiency (190). In the next subsection is presented vitamin B12.

### 5.10 Vitamin B12

The vitamin B complex also includes vitamin B12. This group of eight vitamins helps with everything from maintaining maximum cognitive performance to converting the carbs consumption into the energy that may utilized (191). Vitamin B12, commonly referred to as cobalamin, supports several vital cellular functions. These consist of the synthesis of DNA; the production of healthy red blood cells (192); the growth and functioning of the central nervous system; and the activity of enzymes (193) (**Table 2**). It has been shown that a B12 absence increases the misincorporation of uracil, which restricts DNA synthesis and causes genomic instability. The hypomethylation of DNA, a marker of early carcinogenesis, is another effect of the B12 lack.

In light of the restricted treatment options for canine tumors, companion animal testing for new drugs could lead to the discovery of more effective treatments for oncology in both veterinary and human medicine. Four dogs with spontaneous cancer were used to test the anti-tumor effects of nitrosylcobalamin (NO-Cbl), a vitamin B12-based carrier of nitric oxide (NO) that induces apoptosis. (a) A 13-year-old female sterilized Giant Schnauzer suffering from hypercalcemia and incurable thyroid cancer. (b) A male Golden Retriever, 6 years old, has been sterilized and had a malignant peripheral nerve sheath tumor (MPNST). (c) Anal sac adenocarcinoma (AGACA) in a ten-year-old male Bichon Frise that has been sterilized. (d) A seven-year-old female Labrador endured partial surgical resection for spinal meningioma. The results were: a) After receiving daily NO-Cbl treatment for 10 weeks, the tumor volume in the Giant Schnauzer showed a 77% reduction. (b) After receiving daily NO-Cbl therapy for 15 months, the tumor volume in the Golden Retriever showed a 53% reduction. (c) After 15 months of treatment, the Bichon Frise showed a 43% shrinkage of the main tumor and a 90% regression of an iliac lymph node. The dog’s condition is now stable after 61 months, with normal liver enzymes, a CBC investigation, and no signs of poisoning. (d) After receiving treatment for six months, the Labrador showed a total reduction of the remaining tumor. According to the scientists’ conclusion, using NO-Cbl is a promising anti-cancer therapy that takes advantage of the vitamin B12 receptor’s tumor-specific characteristics (194) (**Table 2**). In what follows, vitamin B13 will be presented.

### 5.11 Vitamin B13

In mammals, orotate (OA) or vitamin B13 is released from the mitochondrial dihydroorotate dehydrogenase (DHODH) and converted to UMP by the cytoplasmic UMP synthase enzyme. OA is well-known as a precursor in the production of pyrimidines. OA is also a common dietary component and can be found in dairy products and milk. It is mostly used by the liver, kidney, and erythrocytes in the pyrimidine salvage pathway when it is converted to uridine. Nutritional studies designated orotate as “vitamin B13” and its application in body-building and metabolic syndrome support has been promoted by its combination with metal ions or organic cations (195). Orotic acid (OA) is an insignificant component of diets and is an intermediate molecule of pyrimidine nucleotide production. Urinary orotic acid testing helps verify the diagnosis of genetic metabolic disorders. Furthermore, understanding how this metabolite’s physiology level changes in connection to other aspects of clinical normalcy may be interesting (196).

According to recent research, magnesium orotate, a combination of magnesium and orotic acid, can be used as an adjuvant treatment for type 2 diabetes, hypertension, congestive heart failure, and post-operative cardiac conditions. Magnesium orotate is superior because it is more easily absorbed, accumulates intracellularly, increases muscle endurance, and even has some antioxidative and antitumor protective properties (197, 198) (**Table 2**). Magnesium orotate has also improved the regeneration of neural tissue shape and reduced nerve cell damage (198, 199). Consequently, the scientific community has begun to formulate theories regarding the use of magnesium orotate in neuropsychiatric diseases. Therefore, more research should be done on any potential links between magnesium orotate, the gut microbiota, and the biochemical equilibrium of the brain.

It is appropriate for studying the various aspects of the small molecule orotic acid (OA), which is widely recognized as a crucial step in the *de novo* synthesis of pyrimidines. Furthermore, erythrocytes and hepatocytes can absorb it and utilize it in the pyrimidine recycling way and for uridine synthesis.

It has been demonstrated that orotate derivatives can be used as anti-pyrimidine medications and in complexes with metal ions and organic cations to support metabolic syndrome treatments. Present genetic research appears to connect reduced orotate production to abnormalities in the dihydroorotate dehydrogenase (DHODH) gene, which causes human Miller syndrome. Different symptoms are associated with orotic aciduria, a disorder of pyrimidine biosynthesis that take place in cattle, and people deficient in UMP synthase (UMPS). Researchers conclude from more results that OA might be involved in controlling gene transcription (200). In the next subsection, the immunomodulatory effects of vitamin B15 will be mentioned.

### 5.12 Vitamin B15-pangamic acid

Since its discovery in 1938, pangamic acid (6-O-(dimethylaminoacetyl)-D-gluconic acid) has been identified as a naturally occurring, widely distributed chemical having a variety of biological and therapeutic uses. In this regard, pangamic acid has been a medication that stimulates cellular respiration for decades on the market throughout the world. Apart from pangamic acid that occurs naturally, di-isopropyl-ammonium dichloroacetate (DIPA), a synthetic product that does not exist in biological material, is available on the market with claims to have comparable biological properties (201). The evolution of dichloroacetate (DCA) as a pharmaceutical agent has a more convoluted history (202). The methylated variants of pangamic acid, also known as d glucono dimethylamino acetate, a supposedly naturally occurring B vitamin, were synthesized in the 1950s using the chemical diisopropylammonium dichloroacetate (DIPA). Pangamate has “the property of eliminating toxic products formed in the human system” and is “a solution for the immunization of toxic products present in the human or animal system,” according to the B15 patent (203). The rate-limiting enzyme of cholesterogenesis, HMG-CoA-reductase, is inhibited noncompetitively by DCA. Additionally, DCA prevents the production of hepatic triglycerides by an unidentified mechanism (204). According to Stacpoole et al. (205) and Moore et al. (206), these mechanisms are responsible for the lipid and lipoprotein-lowering impact of DCA in individuals with diabetes mellitus and the uncommon condition homozygous familial hypercholesterolemia.

In response to elevated serum levels of high-density lipoproteins (HDLP), calcium pangamate (CP) in patients with cerebrasthenic syndrome of atherosclerotic genesis allows a decrease in the cholesterol index of atherogenicity. The activation of phospholipid incorporation into HDLP is one explanation that leads to an increase in HDLP levels in the blood serum of patients under the influence of CP (207). In one trial, 95 patients received fierce treatment (isoniazid, rifampicin, streptomycin, pyrazinamide or ethambutol, vitamins for the first two to three months) out of the 155 patients with damaging pulmonary tuberculosis included in the trial. In addition to one of the antihypoxants (piracetam, calcii pangamas, piriditol), 69 patients representing the other group got adjuvant antioxidants (galascorbin or tocopherol acetate). Intense chemotherapy contributed to bacterial discharge dimension reduction, annihilation discontinuation, cavernous reconstruction, and decreased side effect incidence rates enhanced metabolic processes indicating redox and lipid peroxidation (208).

Either dimethyglycine hydrochloride (DMG) or diisopropylamine dichloroacetate (DIPA-DCA) are present in a large number of compounds marketed as B15 (203). The Ames Salmonella mammalian microsome mutagenicity test (209) was used in recent research to determine the mutagenicity of dimethylglycine incubated with nitrites under conditions that resembled long-term human ingestion (210). As part of diisopropylamine dichloroacetate, dichloroacetate has also been shown to be directly harmful to humans and animals (211), as well as mutagenic in the Ames mutagenicity test (212).

There is also discussion of the numerous myths and poor recommendations around vitamins and health, including “fake” vitamins like pangamic acid (“vitamin B15”) and laetrile (“vitamin B17”). Based on the available data, it would not be appropriate to recommend high-dose vitamin intake or substantial changes to typical vitamin intake levels from a balanced diet as a means to reduce cancer risk in the general population. Nonetheless, a cautious approach to nutrition and eating habits is suggested to improve the health effects associated with our complex lifestyle (213).

In animals, vitamin B15 has specific pharmacological characteristics. These include the generation of hypotension in dogs under anesthesia and neuromuscular blocking action in rabbits and chickens. Neostigmine methylsulfate is an effective antagonist of the neuromuscular blockade produced in the rabbit. These results seem qualitatively comparable to thiamine hydrochloride reports (214). In the next paragraph it will presented vitamin B17.

### 5.13 Vitamin B17

Amygdalin is a cyanogenic glycoside chemical that is mostly found in fruit pulp and kernels. It is sometimes referred to as vitamin B17 and is also a synthetic compound called laetrile. For a long time, this substance has been recommended as a possibly useful naturally generated chemical with anticancer properties.

The use of amygdalin, an artificial substance commonly referred to as vitamin B17 or laetrile, in the prevention and/or co-treatment of cancer is a topic of study. Numerous investigations have exhibited a broad spectrum of biological characteristics for amygdalin, indicating that it could potentially have a preventive or even co-treatment effect on cancers of the bladder, prostate, lung, and cervical regions. This effect could primarily be attributed to the inhibition of cancer cell proliferation (215). The biological activities of amygdalin extracts from three cultivars of cassava (Manihot esculenta) cultivated in Benin were assessed both *in vitro* and *in vivo* (216). The results showed that this naturally occurring molecule may function effectively in co-treatment and cancer prevention by inhibiting the growth of cancer cells.

Thus, amygdalin has been shown *in vitro* experiments to induce apoptosis due to upregulated expression of caspase-3 and Bax protein and downregulated expression of Bcl-2, an anti-apoptotic protein (217) (**Table 2**). Erikel et al. (218) observed that amygdalin may have a modulatory influence on chemotherapeutic drugs that appear to cause genomic damage in human cells, which is relevant to the chemopreventive potential of amygdalin (218). The primary molecular mechanisms of amygdalin’s basic anticancer effects have been attributed to immune function regulation, cytotoxic effect stimulation, apoptosis induction, and cell cycle inhibition in humans (219). More specifically, cellular replication of the cytochrome C Bax protein triggers the activation of the caspase-3 protease, which is the primary molecular mechanism of apoptosis (220). Apoptosis and the ensuing growth of cells have been linked to high expression of the pro-apoptotic protein Bax (221).

As a result, it was postulated that amygdalin causes apoptosis in HeLa cells by elevating caspase-3 and suppressing Bcl-2. At the same time, HeLa cells treated with amygdalin seem to have an elevated Bax level, showing the possibility of an endogenous route facilitating apoptosis (222). Amygdalin has been demonstrated to induce apoptosis and cell cycle arrest in several cell lines from humans, including those from cancer cells of the lung, breast, colon, testes, prostate, rectum, and bladder (219, 223–226). Vitamin B17 was also found to decrease the migration of MDA-MB-231 cells more than MCF-7 cells (227). Furthermore, the inhibition of proteolytic enzymes was suggested to promote the activation of apoptotic events in MCF-7 breast cancer cells (228). In addition, amygdalin was shown to increase Bax and decrease Bcl-2 expression in SK-BR-3 and MCF-7 breast cancer cells. However, compared with the amygdalin–ZHER2 affibody conjugate, the effect on Bax and Bcl-2 expression in SK-BR-3 cells was stronger than that in MCF-7 cells (229).

Amygdalin is frequently utilized for the treatment and prevention of colorectal tumor malignancies in both conventional and alternative medicine (230). It has been observed that vitamin B17 promotes the anticancer impact of the drug by downregulating the expression of genes associated with the cell cycle in colorectal cancer cells, such as human SNU-C4 colorectal cancer cells (231). Due to their higher concentration of β-glucosidase and lower levels of the liver enzyme rhodanese, which can convert cyanide to the comparatively innocuous chemical thiocyanate, colon cancer cells have been reported to be more vulnerable to the impact of amygdalin compared to normal cells (232). Through increasing caspase-3 expression and decreasing Bcl-2 expression, as well as by reducing the proliferation of HepG2 and EAC hepatocellular carcinoma cells and upregulating Beclin-1 expression, amygdalin has been shown to induce the apoptotic process (233). It’s noteworthy to note that the combination of amygdalin and metformin showed promise in terms of cytotoxicity and ability to induce apoptosis and stop the cell cycle in hepatocellular carcinoma cells when compared to amygdalin alone (234). Apart from this mixture, it has also been demonstrated that the action of amygdalin with zinc has a greater apoptotic impact in the therapy of HepG2 than does amygdalin alone (235).

In animals, the maximum dosage of amygdalin that, when administered intramuscularly and intravenously to mice, rabbits, and dogs, did not result in any intolerable side effects was 3 g/kg; orally, the dose was 0.075 g/kg. Additionally, the maximum tolerated dose of amygdalin administered intravenously to humans was around 0.07 g/kg. According to several studies, amygdalin can selectively target cancer cells while sparing healthy cells, lower telomerase activity, and stop the proliferation of bladder cancer cells. However, cyanide compounds produced during amygdalin decomposition caused important side effects when amygdalin was utilized (236) (**Table 2**).

**Figure 3:**
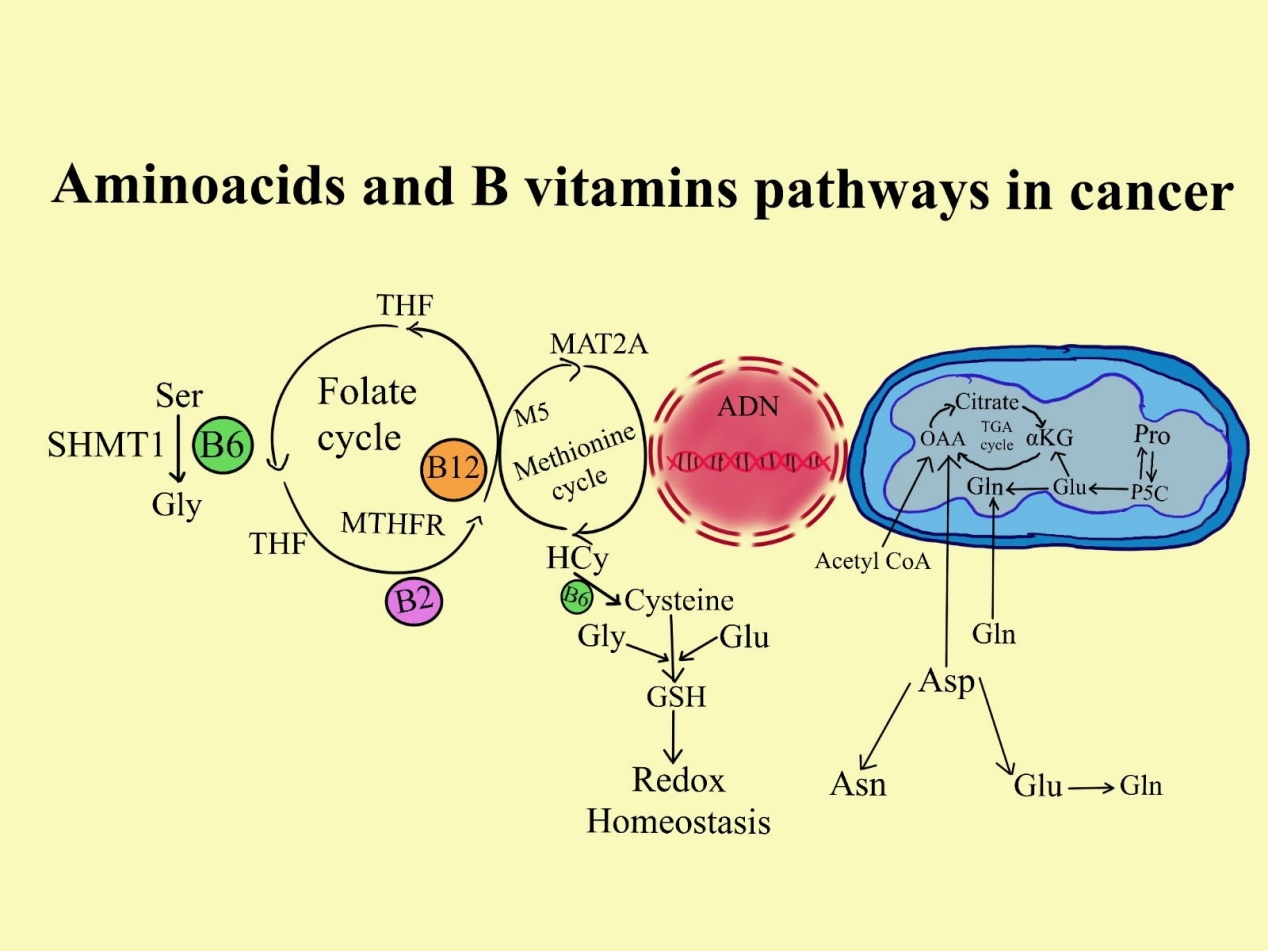
The involvement of B vitamins in different cancer cell metabolism. Through the citric acid cycle, amino acids generate metabolic intermediates like acetyl-CoA that support energy generation. Additionally, amino acids supply the building blocks for lipogenesis and nucleotide synthesis, two processes essential to a cell's capacity for growth and development.

As mentioned, a strong association exists between cancer cells and reactive oxygen species. Reactive oxygen species grow as a result of cancer cell proliferation, which may damage macromolecules and ultimately cause cell death. To maintain redox balance, cancer cells must synthesize glutathione from glutamate, glycine, and cysteine (237). Although the pentose phosphate pathway and glycolysis are well recognized as the primary sources of NADPH that contribute to redox balance, the folate cycle (**Figure 3**)—which is mostly sustained by serine-derived one-carbon units—also generates a significant amount of NADPH (238). Along with their direct involvement in metabolic reprogramming processes, amino acids, and their derivatives play a crucial role in mediating epigenetic regulation and posttranscriptional modification.

In particular, normal metabolite levels in the methionine cycle, which are affected by methionine, serine, and glycine modulate DNA and histone methylation (239). Similarly, acetyl-CoA, which results from different metabolic pathways, is needed for histone acetylation, processes that promote gene expression and the advancement of cancer. Acetyl-CoA produced from amino acids is also important for acetylating proteins, which may contribute to the formation of tumors (240) (**Figure 3**).

By producing glutathione, amino acids can control redox balance and evade the consequences of oxidative stress. Tricarboxylic acid cycle (TCA); Alpha-ketoglutaric acid (α-KG); pyrroline-5-carboxylate (P5C) glutathione (GSH); acetyl-coenzyme A (Acetyl-coA); Aspartate transaminase (ASP); Methylenetetrahydrofolate reductase (MTHFR); Serine hydroxymethyltransferase 1 (SHMT1); methionine adenosyltransferase II alpha (MAT2A); Tetrahydrofolate (THF); Asparagine (ASN), Arginine (ARG).

## 6 Conclusions and future perspective

As mentioned, there is a close connection between the nutrients in the diet and the immune system. In humans, this connection is intensively studied, which does not happen in the case of animals. Most studies regarding nutritional requirements are extrapolated from humans to animals. This is sad, considering the particularity of the processes of digestion and absorption that ultimately make a difference between the effects caused to humans and those of animals. It was shown that different micronutrients fat-soluble and water-soluble vitamins affect immunity through biochemical, genetic, and signaling pathways. Additionally, the novelty and complexity of this review consist in the fact that it treats all vitamins and everything known about their immunological effects in both humans and animals.

Furthermore, this paper offers findings indicating the potential role of several micronutrients, in the prevention and treatment of immune-related disorders. Because of this, eating free-fat (light) foods essentially deprives mammals of fat-soluble vitamins and some precursors for active compounds, which impact immune system function. Therefore, diets should include the right class of compounds in the right amounts. Otherwise, the foods that are consumed would be depleted in nutrients. Furthermore, some nutrients such as fat-soluble vitamins improve the flavor and satiety of food significantly. Also, it emphasized interspecies differences and the fact that the response in pathophysiologic stages is significantly different from normal physiologic stages. The immunological response is hence unique and unpredictable (88, 241). In addition, the possible roles that specific vitamins may play in cancer, given their functions as cofactors in processes related to energy and proliferation that ultimately result in the growth of tumor cells were presented.

1. Therefore, pro-apoptotic proteins can be activated and anti-apoptotic proteins can be suppressed by calcitriol/VDR signaling, which may also reduce tumor-associated inflammation by suppressing the cyclooxygenase-2, prostaglandin, and NF-kB pathways. Consequently, all previous mechanisms cooperate to suppress the growth of tumors (42).
2. Due to oxidative stress’s role in the development of cancer, α-T’s ability to prevent cancer has been thoroughly researched. A higher cancer risk has been associated with reduced VE intake or nutritional status. Certain intervention studies have shown that treating with α-T has a favorable effect on minimizing cancer risk when VE deficiency is present.
3. Vitamin K in the aberrant form known as PIVKA-II is elevated in certain neoplastic illnesses and human coagulation dysfunctions.
4. Vitamin C has been connected with the prevention, progression, and treatment of cancer (76).
5. Research on the connection between vitamin C and cancer is ongoing. Differences in results are due to a combination of factors including ascorbic acid-related concerns (doses, administration routes, plasmatic levels, metabolites recording, and source), cancer characteristics (specific mutations present, type and grade of cancer, received conventional anti-cancer therapy), and individual characteristics (diet, behaviors, genetics, transporters). This connects the recording of doses to an active anti-cancer mechanism and makes them effective (76).
6. A lack of vitamin B2 is linked to anemia, neurotoxicity, growth retardation, and potentially some types of cancer (105). Indeed, it has recently been demonstrated that riboflavin lack increases cell proliferation and decreases cell viability in HepG2 cells (106).
7. The incidence of CRC was inversely associated with vitamin B6 intake and blood PLP levels (131). According to Ames and Wakimoto (132), PLP has been suggested to impact carcinogenesis *via* a variety of mechanisms, including those involving DNA metabolism.
8. In primary human lymphocytes, folate deprivation decreased cell proliferation and increased apoptosis, cell cycle arrest, and DNA strand damage (165). Some research found that compared to aged corresponding controls, newly diagnosed cancer patients had mean levels of homocysteine significantly higher and vitamin B9 levels significantly lower. This suggests that low folate and high homocysteine may be linked to lung cancer (166).
9. PABA/B10 and radiation treatment together accelerated the death of tumor cells.
10. Vitamin B17 was also found to decrease the migration of MDA-MB-231 cells more than MCF-7 cells (227). In addition, amygdalin was shown to increase Bax and decrease Bcl-2 expression in SK-BR-3 and MCF-7 breast cancer cells.

Regarding the immunomodulatory effects of all nutrients, it is good to take into account the recommended daily dose. As can be seen, the values outside the Gauss curve are not beneficial for the mammalian body. Of course, this is valid after the recommended daily dose is established, here it is referring to animals. Moreover, we hope that through the information we have provided, guidelines and strategies can be built to prevent and improve the lives of four-legged cancer patients. This article shows that there is a need to carry out new studies on the particularities of digestion and their influence on neoplasias, showing the difference between animals and humans.

## References

[1] Coates M Blanchard S MacLeod AS. Innate antimicrobial immunity in the skin: A protective barrier against bacteria, viruses, and fungi. PloS pathogens. (2018) 14:e1007353. doi: 10.1371/journal.ppat.1007353 30522130 PMC6283644

[2] Hébuterne X Lemarié E Michallet M de Montreuil CB Schneider SM Goldwasser F. Prevalence of malnutrition and current use of nutrition support in patients with cancer. J Parenteral Enteral Nutr. (2014) 38:196–204. doi: 10.1177/0148607113502674 24748626

[3] Ottery FD. Supportive nutrition to prevent cachexia and improve quality of life. Semin Oncol. (1995) 22:98–111. 7740324

[4] Baracos VE Martin L Korc M Guttridge DC Fearon KC. Cancer-associated cachexia. Nat Rev Dis primers. (2018) 4:1–18. doi: 10.1038/nrdp.2017.105 29345251

[5] Molfino A Iannace A Colaiacomo MC Farcomeni A Emiliani A Gualdi G. Cancer anorexia: hypothalamic activity and its association with inflammation and appetite-regulating peptides in lung cancer. J cachexia sarcopenia muscle. (2017) 8:40–7. doi: 10.1002/jcsm.12156 PMC5326827 27897393

[6] Cederholm T Morley JE. Nutrient interface with biology and aging. Curr Opin Clin Nutr Metab Care. (2016) 19:1–4. doi: 10.1097/MCO.0000000000000241 26560526

[7] Barber L Weishaar K. Criteria for designation of clinical substage in canine lymphoma: a survey of veterinary oncologists. Vet Comp Oncol. (2016) 14:32–9. doi: 10.1111/vco.12086 27508350

[8] Johannes CM Musser ML. Anorexia and the cancer patient. Vet Clinics: Small Anim Practice. (2019) 49:837–54. doi: 10.1016/j.cvsm.2019.04.008 31176457

[9] Rhodes L Zollers B Wofford JA Heinen E. Capromorelin: a ghrelin receptor agonist and novel therapy for stimulation of appetite in dogs. Vet Med Sci. (2018) 4:3–16. doi: 10.1002/vms3.83 29468076 PMC5813110

[10] Howick K Griffin BT Cryan JF Schellekens H. From belly to brain: targeting the ghrelin receptor in appetite and food intake regulation. Int J Mol Sci. (2017) 18:273. doi: 10.3390/ijms18020273 28134808 PMC5343809

[11] Kojima M Kangawa K. Ghrelin: structure and function. Physiol Rev. (2005) 85:495–522. doi: 10.1152/physrev.00012.2004 15788704

[12] Cummings DE Frayo RS Marmonier C Aubert R Chapelot D. Plasma ghrelin levels and hunger scores in humans initiating meals voluntarily without time-and food-related cues. Am J Physiology-Endocrinol Metab. (2004) 287:E297–304. doi: 10.1152/ajpendo.00582.2003 15039149

[13] Bhatti SF Hofland LJ van Koetsveld PM Van Ham LM Duchateau L Mol JA. Effects of food intake and food withholding on plasma ghrelin concentrations in healthy dogs. Am J vet Res. (2006) 67:1557–63. doi: 10.2460/ajvr.67.9.1557 16948601

[14] Ida T Miyazato M Naganobu K Nakahara K Sato M Lin X-Z. Purification and characterization of feline ghrelin and its possible role. Domest Anim endocrinol. (2007) 32:93–105. doi: 10.1016/j.domaniend.2006.01.002 16466902

[15] Molon-Noblot S Laroque P Prahalada S Stabinski LG Hoe C-M Peter CP. Effect of chronic growth hormone administration on skeletal muscle in dogs. Toxicol pathol. (1998) 26:207–12. doi: 10.1177/019262339802600203 9547857

[16] Temel JS Abernethy AP Currow DC Friend J Duus EM Yan Y. Anamorelin in patients with non-small-cell lung cancer and cachexia (ROMANA 1 and ROMANA 2): results from two randomised, double-blind, phase 3 trials. Lancet Oncol. (2016) 17:519–31. doi: 10.1016/S1470-2045(15)00558-6 26906526

[17] Donaldson MS. Nutrition and cancer: a review of the evidence for an anti-cancer diet. Nutr J. (2004) 3:19. doi: 10.1186/1475-2891-3-19 15496224 PMC526387

[18] Ogilvie GK Fettman MJ Mallinckrodt CH Walton JA Hansen RA Davenport DJ. Effect of fish oil, arginine, and doxorubicin chemotherapy on remission and survival time for dogs with lymphoma: a double-blind, randomized placebo-controlled study. Cancer: Interdiscip Int J Am Cancer Soc. (2000) 88:1916–28. doi: 10.1002/(ISSN)1097-0142 10760770

[19] Geng P Qin W Xu G. Proline metabolism in cancer. Amino Acids. (2021) 53:1769–77. doi: 10.1007/s00726-021-03060-1 34390414

[20] Kabir Y. . Functional foods in cancer prevention and therapy. London, United Kingdom; San Diego, CA, United States; Cambridge, MA, United States; Oxford, United Kingdom: Academic Press (2020).

[21] Pham VT Dold S Rehman A Bird JK Steinert RE. Vitamins, the gut microbiome and gastrointestinal health in humans. Nutr Res. (2021) 95:35–53. doi: 10.1016/j.nutres.2021.09.001 34798467

[22] Barko PC Williams DA. Serum concentrations of lipid-soluble vitamins in dogs with exocrine pancreatic insufficiency treated with pancreatic enzymes. J vet Internal Med. (2018) 32:1600–8. doi: 10.1111/jvim.15292 PMC6189355 30133868

[23] Hall EJ. Small intestine. In: Canine and feline gastroenterology. Bristol, United Kingdom: JAI-Elsevier Science Inc (2012). p. 651–728.

[24] Villamor E Fawzi WW. Effects of vitamin A supplementation on immune responses and correlation with clinical outcomes. Clin Microbiol Rev. (2005) 18:446–64. doi: 10.1128/CMR.18.3.446-464.2005 PMC1195969 16020684

[25] Maggini S Beveridge S Sorbara PJ Senatore G. Feeding the immune system: the role of micronutrients in restoring resistance to infections. CABI Rev. (2008) 2009:1–21. doi: 10.1079/PAVSNNR20083098

[26] Cox S Arthur P Kirkwood B Yeboah-Antwi K Riley E. Vitamin A supplementation increases ratios of proinflammatory to anti-inflammatory cytokine responses in pregnancy and lactation. Clin Exp Immunol. (2006) 144:392–400. doi: 10.1111/j.1365-2249.2006.03082.x 16734607 PMC1941972

[27] Papiernik D Urbaniak A Kłopotowska D Nasulewicz-Goldeman A Ekiert M Nowak M. Retinol-binding protein 4 accelerates metastatic spread and increases impairment of blood flow in mouse mammary gland tumors. Cancers. (2020) 12:623. doi: 10.3390/cancers12030623 32156052 PMC7139568

[28] Schedlbauer C Blaue D Raila J Vervuert I. Alterations of serum vitamin E and vitamin A concentrations of ponies and horses during experimentally induced obesity. J Anim Physiol Anim Nutr. (2020) 104:1501–8. doi: 10.1111/jpn.13385 32406587

[29] Farjo KM Farjo RA Halsey S Moiseyev G Ma J-x. Retinol-binding protein 4 induces inflammation in human endothelial cells by an NADPH oxidase-and nuclear factor kappa B-dependent and retinol-independent mechanism. Mol Cell Biol. (2012) 32:5103–15. doi: 10.1128/MCB.00820-12 PMC3510526 23071093

[30] Tang D Tao D Fang Y Deng C Xu Q Zhou J. TNF-alpha promotes invasion and metastasis via NF-kappa B pathway in oral squamous cell carcinoma. Med Sci monitor basic Res. (2017) 23:141. doi: 10.12659/MSMBR.903910 PMC5391804 28386055

[31] Xie T-X Xia Z Zhang N Gong W Huang S. Constitutive NF-κB activity regulates the expression of VEGF and IL-8 and tumor angiogenesis of human glioblastoma. Oncol Rep. (2010) 23:725–32. doi: 10.3892/or_00000690 20127012

[32] Gil Á Plaza-Diaz J Mesa MD. Vitamin D: classic and novel actions. Ann Nutr Metab. (2018) 72:87–95. doi: 10.1159/000486536 29346788

[33] Ryan JW Anderson PH Morris HA. Pleiotropic activities of vitamin D receptors–adequate activation for multiple health outcomes. Clin Biochem Rev. (2015) 36:53. 26224895 PMC4504155

[34] Shin M-H Holmes MD Hankinson SE Wu K Colditz GA Willett WC. Intake of dairy products, calcium, and vitamin D and risk of breast cancer. J Natl Cancer Instit. (2002) 94:1301–10. doi: 10.1093/jnci/94.17.1301 12208895

[35] Jacobson EA. Effects of dietary fat, calcium and vitamin D on growth and mammary tumorigenesis induced by 7, 12-dimeethylbenzo (a) anthracene in female Sprague-Dawley rats. Cancer Res. (1989) 49:6300–3. 2509066

[36] Mehta R Hawthorne M Uselding L Albinescu D Moriarty R Christov K. Prevention of N-methyl-N-nitrosourea-induced mammary carcinogenesis in rats by 1α-hydroxyvitamin D5. J Natl Cancer Instit. (2000) 92:1836–40. doi: 10.1093/jnci/92.22.1836 11078761

[37] Anzano MA Smith JM Uskoković MR Peer CW Mullen LT Letterio JJ. 1α, 25-Dihydroxy-16-ene-23-yne-26, 27-hexafluorocholecalciferol (Ro24-5531), a new deltanoid (vitamin D analogue) for prevention of breast cancer in the rat. Cancer Res. (1994) 54:1653–6. 8137276

[38] Morris JG. Ineffective vitamin D synthesis in cats is reversed by an inhibitor of 7-dehydrocholestrol-Δ7-reductase. J Nutr. (1999) 129:903–8. doi: 10.1093/jn/129.4.903 10203568

[39] Morris JG. Idiosyncratic nutrient requirements of cats appear to be diet-induced evolutionary adaptations. Nutr Res Rev. (2002) 15:153–68. doi: 10.1079/NRR200238 19087402

[40] Zafalon RV Risolia LW Pedrinelli V Vendramini TH Rodrigues RB Amaral AR. Vitamin D metabolism in dogs and cats and its relation to diseases not associated with bone metabolism. J Anim Physiol Anim Nutr. (2020) 104:322–42. doi: 10.1111/jpn.13259 31803981

[41] Davies J Heeb H Garimella R Templeton K Pinson D Tawfik O. Vitamin D receptor, retinoid X receptor, Ki-67, survivin, and ezrin expression in canine osteosarcoma. Vet Med Int. (2012) 2012:761034. doi: 10.1155/2012/761034 23346460 PMC3544330

[42] Welsh J. Function of the vitamin D endocrine system in mammary gland and breast cancer. Mol Cell endocrinol. (2017) 453:88–95. doi: 10.1016/j.mce.2017.04.026 28579119 PMC5538720

[43] Munné-Bosch S. [amp]]alpha;-Tocopherol: a multifaceted molecule in plants. Vit Hormones. (2007) 76:375–92. doi: 10.1016/S0083-6729(07)76014-4 17628182

[44] Niki E Abe K. Vitamin E: Structure, properties and functions. Food Chemistry, Function and Analysis No.11 Vitamin E: Chemistry and Nutritional Benefits. (2019). doi: 10.1039/2398-0664

[45] Munteanu C Berindean I Mihai M Pop B Popa M Muntean L. E, K, B5, B6, and B9 vitamins and their specific immunological effects evaluated by flow cytometry. Front Med. (2023) 9:1089476. doi: 10.3389/fmed.2022.1089476 PMC9849766 36687400

[46] Jayedi A Rashidy-Pour A Parohan M Zargar MS Shab-Bidar S. Dietary and circulating vitamin C, vitamin E, β-carotene and risk of total cardiovascular mortality: A systematic review and dose–response meta-analysis of prospective observational studies. Public Health Nutr. (2019) 22:1872–87. doi: 10.1017/S1368980018003725 PMC10260571 30630552

[47] Traber MG Jialal I. Measurement of lipid-soluble vitamins-further adjustment needed? Lancet. (2000) 355:2013–4. doi: 10.1016/S0140-6736(00)02345-X 10885350

[48] Yang CS Luo P Zeng Z Wang H Malafa M Suh N. Vitamin E and cancer prevention: Studies with different forms of tocopherols and tocotrienols. Mol carcinogen. (2020) 59:365–89. doi: 10.1002/mc.23160 PMC7255062 32017273

[49] Hattori S Hattori Y Banba N Kasai K Shimoda S. Pentamethyl-hydroxychromane, vitamin E derivative, inhibits induction of nitric oxide synthase by bacterial lipopolysaccharide. Biochem Mol Biol Int. (1995) 35:177–83. 7537570

[50] Devaraj S Li D Jialal I. The effects of alpha tocopherol supplementation on monocyte function. Decreased lipid oxidation, interleukin 1 beta secretion, and monocyte adhesion to endothelium. J Clin Invest. (1996) 98:756–63. doi: 10.1172/JCI118848 PMC507486 8698868

[51] Ford ES Mokdad AH Giles WH Brown DW. The metabolic syndrome and antioxidant concentrations: findings from the Third National Health and Nutrition Examination Survey. Diabetes. (2003) 52:2346–52. doi: 10.2337/diabetes.52.9.2346 12941775

[52] Rhouma M de Oliveira El Warrak A Troncy E Beaudry F Chorfi Y. Anti-inflammatory response of dietary vitamin E and its effects on pain and joint structures during early stages of surgically induced osteoarthritis in dogs. Can J Vet Res. (2013) 77:191–8. PMC3700444 24101795

[53] Namazi N Larijani B Azadbakht L. Vitamin K and the immune system. Nutr Immun. (2019) 3:75–9. doi: 10.1007/978-3-030-16073-9_4

[54] Joyce DE Grinnell BW. Recombinant human activated protein C attenuates the inflammatory response in endothelium and monocytes by modulating nuclear factor-κB. Crit Care Med. (2002) 30:S288–S93. doi: 10.1097/00003246-200205001-00019 12004250

[55] O’Neil J Scarrott B Svalheim RA Elliott J Hodges SJ. Vitamin K2 in animal health: an overview. In: Gordeladze JO, editor. Vitamin K2-Vital for Health and Wellbeing. INTECH (2017) p. 215–236.

[56] Plaza SM Lamson ND. The anticancer effects of vitamin K. Altern Med review. (2003) 8:303–18. 12946240

[57] Shearer MJ Bach A Kohlmeier M. Chemistry, nutritional sources, tissue distribution and metabolism of vitamin K with special reference to bone health. J Nutr. (1996) 126:1181S–6S. doi: 10.1093/jn/126.suppl_4.1181S 8642453

[58] Tokita H Tsuchida A Miyazawa K Ohyashiki K Katayanagi S Sudo H. Vitamin K2-induced antitumor effects via cell-cycle arrest and apoptosis in gastric cancer cell lines. Int J Mol Med. (2006) 17:235–43. doi: 10.3892/ijmm 16391821

[59] Verrax J Cadrobbi J Delvaux M Jamison JM Gilloteaux J Summers JL. The association of vitamins C and K3 kills cancer cells mainly by autoschizis, a novel form of cell death. Basis for their potential use as coadjuvants in anticancer therapy. Eur J med Chem. (2003) 38:451–7. doi: 10.1016/S0223-5234(03)00082-5 12767595

[60] Von Gruenigen VE Jamison JM Gilloteaux J Lorimer HE Summers M Pollard RR. The in *vitro* antitumor activity of vitamins C and K3 against ovarian carcinoma. Anticancer Res. (2003) 23:3279–87. 12926064

[61] Yokoyama T Miyazawa K Yoshida T Ohyashiki K. Combination of vitamin K2 plus imatinib mesylate enhances induction of apoptosis in small cell lung cancer cell lines. Int J Oncol. (2005) 26:33–40. doi: 10.3892/ijo 15586222

[62] Yoshida T Miyazawa K Kasuga I Yokoyama T Minemura K Ustumi K. Apoptosis induction of vitamin K2 in lung carcinoma cell lines: the possibility of vitamin K2 therapy for lung cancer. Int J Oncol. (2003) 23:627–32. doi: 10.3892/ijo 12888897

[63] Jamison JM Gilloteaux J Taper HS Summers JL. Evaluation of the in *vitro* and in *vivo* antitumor activities of vitamin C and K-3 combinations against human prostate cancer. J Nutr. (2001) 131:158S–60S. doi: 10.1093/jn/131.1.158S 11208954

[64] Ogawa M Nakai S Deguchi A Nonomura T Masaki T Uchida N. Vitamins K2, K3 and K5 exert antitumor effects on established colorectal cancer in mice by inducing apoptotic death of tumor cells. Int J Oncol. (2007) 31:323–31. doi: 10.3892/ijo 17611688

[65] Osada S Saji S Osada K. Critical role of extracellular signal-regulated kinase phosphorylation on menadione (vitamin K3) induced growth inhibition. Cancer: Interdiscip Int J Am Cancer Soc. (2001) 91:1156–65. doi: 10.1002/(ISSN)1097-0142 11267961

[66] Wu FY-H Liao W-C Chang H-M. Comparison of antitumor activity of vitamins K1, K2 and K3 on human tumor cells by two (MTT and SRB) cell viability assays. Life Sci. (1993) 52:1797–804. doi: 10.1016/0024-3205(93)90469-J 8492642

[67] Welsh J Bak MJ Narvaez CJ. New insights into vitamin K biology with relevance to cancer. Trends Mol Med. (2022) 10:864–81. doi: 10.1016/j.molmed.2022.07.002 PMC9509427 36028390

[68] Lobo V Patil A Phatak A Chandra N. Free radicals, antioxidants and functional foods: Impact on human health. Pharmacog Rev. (2010) 4:118. doi: 10.4103/0973-7847.70902 PMC3249911 22228951

[69] Card DJ Gorska R Harrington DJ. Laboratory assessment of vitamin K status. J Clin Pathol. (2020) 73:70–5. doi: 10.1136/jclinpath-2019-205997 31862867

[70] Kim JM Kwon C Joh J-W Park JB Lee JH Kim SJ. PIVKA-II is a useful marker in patients with modified UICC T3 stage hepatocellular carcinoma. Hepato-gastroenterology. (2013) 60:1456–62. doi: 10.5754/hge13058 23567987

[71] Maniscalco L Varello K Morello E Montemurro V Olimpo M Giacobino D. Investigating a prognostic factor for canine hepatocellular carcinoma: analysis of different histological grading systems and the role of PIVKA-II. Vet Sci. (2022) 9:689. doi: 10.3390/vetsci9120689 36548850 PMC9781990

[72] Wheatley C. The return of the Scarlet Pimpernel: cobalamin in inflammation II—cobalamins can both selectively promote all three nitric oxide synthases (NOS), particularly iNOS and eNOS, and, as needed, selectively inhibit iNOS and nNOS. J Nutr Environ Med. (2007) 16:181–211. doi: 10.1080/10520290701791839 18836533 PMC2556189

[73] Cote E. Clinical veterinary advisor-E-book: Dogs and cats. St. Louis, Missouri, USA: Elsevier Health Sciences (2010).

[74] Chawla J Kvarnberg D. Hydrosoluble vitamins. In: Biller J Ferro JM, editors. Handbook of clinical neurology, vol. 120. (2014). p. 891–914. 10.1016/B978-0-7020-4087-0.00059-0 24365359

[75] Paciolla C Fortunato S Dipierro N Paradiso A De Leonardis S Mastropasqua L. Vitamin C in plants: from functions to biofortification. Antioxidants. (2019) 8:519. doi: 10.3390/antiox8110519 31671820 PMC6912510

[76] Villagran M Ferreira J Martorell M Mardones L. The role of vitamin C in cancer prevention and therapy: a literature review. Antioxidants. (2021) 10:1894. doi: 10.3390/antiox10121894 34942996 PMC8750500

[77] Bsoul SA Terezhalmy GT. Vitamin C in health and disease. J Contemp Dent Pract. (2004) 5:1–13. doi: 10.5005/jcdp-5-2-1 15150630

[78] Avello M Suwalsky M. Radicales libres, antioxidantes naturales y mecanismos de protección. Atenea (Concepción). (2006) 494:161–72. doi: 10.4067/S0718-04622006000200010

[79] Garcia EIC Elghandour MM Khusro A Alcala-Canto Y Tirado-González DN Barbabosa-Pliego A. Dietary supplements of vitamins E, C, and β-carotene to reduce oxidative stress in horses: an overview. J Equine Vet Sci. (2022) 110:103863. doi: 10.1016/j.jevs.2022.103863 35017039

[80] Yoshii K Hosomi K Sawane K Kunisawa J. Metabolism of dietary and microbial vitamin B family in the regulation of host immunity. Front Nutr. (2019) 6:48. doi: 10.3389/fnut.2019.00048 31058161 PMC6478888

[81] Chen AC Martin AJ Choy B Fernández-Peñas P Dalziell RA McKenzie CA. A phase 3 randomized trial of nicotinamide for skin-cancer chemoprevention. New Engl J Med. (2015) 373:1618–26. doi: 10.1056/NEJMoa1506197 26488693

[82] Buque A Bloy N Petroni G Kroemer G Galluzzi L. NK cells beat T cells at early breast cancer control. Taylor Francis;. (2020) p:1806010. doi: 10.1080/2162402X.2020.1806010 PMC7458610 32923169

[83] Selvanesan BC Meena K Beck A Meheus L Lara O Rooman I. Nicotinamide combined with gemcitabine is an immunomodulatory therapy that restrains pancreatic cancer in mice. J immunother Cancer. (2020) 8:001250. doi: 10.1101/2020.06.02.129809 PMC7646363 33154149

[84] Davidson M. Thiamin deficiency in a colony of cats. Vet Rec. (1992) 130:94–7. doi: 10.1136/vr.130.5.94 1557878

[85] Meléndez RR. Importance of water-soluble vitamins as regulatory factors of genetic expression. Rev investig clinica; organo del Hosp Enfermedades la Nutricion. (2002) 54:77–83. 11995411

[86] Trebukhina R Tumanov V Avdechik L Bobko I Tsabenko I. Transketolase activity and thiamine diphosphate content in oncological patients. Voprosy meditsinskoi khimii. (1983) 29:100–3. 6649520

[87] Council NR. Nutrient requirements of dogs and cats. Washington, DC, USA: National Academies Press (2006).

[88] Munteanu C Schwartz B. The relationship between nutrition and the immune system. Front Nutr. (2022) 9:1082500. doi: 10.3389/fnut.2022.1082500 36570149 PMC9772031

[89] Lu’o’ng KVQ Nguyễn LTH. The role of thiamine in cancer: possible genetic and cellular signaling mechanisms. Cancer Genomics proteomics. (2013) 10:169–85. 23893925

[90] Kaaks R Tuyns AJ Haelterman M Riboli E. Nutrient intake patterns and gastric cancer risk: A case-control study in Belgium. Int J cancer. (1998) 78:415–20. doi: 10.1002/(ISSN)1097-0215 9797127

[91] Nesterova V Chebotareva M. Thiamine content and enzyme activity in blood cells in leukemia. Voprosy meditsinskoi khimii. (1976) 22:732–5. 829192

[92] Basu T Dickerson J. The thiamin status of early cancer patients with particular reference to those with breast and bronchial carcinomas. Oncology. (1976) 33:250–2. doi: 10.1159/000225157 1026857

[93] Mansoorabadi SO Thibodeaux CJ Liu H-w. The diverse roles of flavin coenzymes nature’s most versatile thespians. J organic Chem. (2007) 72:6329–42. doi: 10.1021/jo0703092 PMC2519020 17580897

[94] Saedisomeolia A Ashoori M. Riboflavin in human health: a review of current evidences. Adv Food Nutr Res. (2018) 83:57–81. doi: 10.1016/bs.afnr.2017.11.002 29477226

[95] Rao PN Levine E Myers MO Prakash V Watson J Stolier A. Elevation of serum riboflavin carrier protein in breast cancer. Cancer Epidemiol Biomarkers Prev. (1999) 8:985–90. 10566553

[96] Munteanu C Schwartz B. B vitamins, glucoronolactone and the immune system: bioavailability, doses and efficiency. Nutrients. (2023) 16:24. doi: 10.3390/nu16010024 38201854 PMC10780850

[97] Papadimitriou N Bouras E Van den Brandt PA Muller DC Papadopoulou A Heath AK. A prospective diet-wide association study for risk of colorectal cancer in EPIC. Clin Gastroenterol hepatol. (2022) 20:864–73. e13. doi: 10.1016/j.cgh.2021.04.028 33901663

[98] Zschäbitz S Cheng T-YD Neuhouser ML Zheng Y Ray RM Miller JW. B vitamin intakes and incidence of colorectal cancer: results from the Women’s Health Initiative Observational Study cohort. Am J Clin Nutr. (2013) 97:332–43. doi: 10.3945/ajcn.112.034736 PMC3545682 23255571

[99] Morita M Yin G Yoshimitsu S-i Ohnaka K Toyomura K Kono S. Folate-related nutrients, genetic polymorphisms, and colorectal cancer risk: the fukuoka colorectal cancer study. Asian Pacific J Cancer Prev. (2013) 14:6249–56. doi: 10.7314/APJCP.2013.14.11.6249 24377513

[100] Li S Ye J Lin Z Lin Z Tang X Rao W. Dietary inflammatory nutrients and esophageal squamous cell carcinoma risk: A case-control study. Nutrients. (2022) 14:5179. doi: 10.3390/nu14235179 36501209 PMC9737973

[101] Sun Z Zhu Y Wang PP Roebothan B Zhao J Zhao J. Reported intake of selected micronutrients and risk of colorectal cancer: results from a large population-based case–control study in Newfoundland, Labrador and Ontario, Canada. Anticancer Res. (2012) 32:687–96. 22287764

[102] Shrubsole M Yang G Gao Y Chow W Shu X Cai Q. Correction: Dietary B vitamin and methionine intakes and plasma folate are not associated with colorectal cancer risk in Chinese women. Cancer epidemiol Biomarkers Prev. (2012) 21:1003–6. doi: 10.1158/1055-9965.EPI-08-1200 PMC2677023 19240230

[103] de Vogel S Bongaerts BW Wouters KA Kester AD Schouten LJ de Goeij AF. Associations of dietary methyl donor intake with MLH1 promoter hypermethylation and related molecular phenotypes in sporadic colorectal cancer. Carcinogenesis. (2008) 29:1765–73. doi: 10.1093/carcin/bgn074 18339680

[104] Ma Y Huangfu Y Deng L Wang P Shen L Zhou Y. High serum riboflavin is associated with the risk of sporadic colorectal cancer. Cancer Epidemiol. (2023) 83:102342. doi: 10.1016/j.canep.2023.102342 36863217

[105] Thakur K Tomar SK Brahma B De S. Screening of riboflavin-producing lactobacilli by a polymerase-chain-reaction-based approach and microbiological assay. J Agric Food Chem. (2016) 64:1950–6. doi: 10.1021/acs.jafc.5b06165 26902872

[106] Liu D Ke Z Luo J. Thiamine deficiency and neurodegeneration: the interplay among oxidative stress, endoplasmic reticulum stress, and autophagy. Mol neurobiol. (2017) 54:5440–8. doi: 10.1007/s12035-016-0079-9 PMC5337452 27596507

[107] van Herwaarden AE Wagenaar E Merino G Jonker JW Rosing H Beijnen JH. Multidrug transporter ABCG2/breast cancer resistance protein secretes riboflavin (vitamin B2) into milk. Mol Cell Biol. (2007) 27:1247–53. doi: 10.1128/MCB.01621-06 PMC1800714 17145775

[108] Qin Y Zhou J Xiong X Huang J Li J Wang Q. Effect of riboflavin on intestinal development and intestinal epithelial cell function of weaned piglets. J Anim Physiol Anim Nutr. (2023) 107:518–28. doi: 10.1111/jpn.13725 35534939

[109] Williams E Rumsey R Powers H. An investigation into the reversibility of the morphological and cytokinetic changes seen in the small intestine of riboflavin deficient rats. Gut. (1996) 39:220–5. doi: 10.1136/gut.39.2.220 PMC1383302 8991860

[110] Yates CA Evans GS Powers HJ. Riboflavin deficiency: early effects on post-weaning development of the duodenum in rats. Br J Nutr. (2001) 86:593–9. doi: 10.1079/BJN2001420 11737957

[111] Migliavacca E Tay SK Patel HP Sonntag T Civiletto G McFarlane C. Mitochondrial oxidative capacity and NAD+ biosynthesis are reduced in human sarcopenia across ethnicities. Nat Commun. (2019) 10:5808. doi: 10.1038/s41467-019-13694-1 31862890 PMC6925228

[112] Badawy AA Guillemin GJ. Species differences in tryptophan metabolism and disposition. Int J Tryptophan Res. (2022) 15:11786469221122511. doi: 10.1177/11786469221122511 36325027 PMC9620070

[113] Braund K. Clinical Neurology in Small Animals-Localization, Diagnosis and Treatment. Braund KG, editor. USA: Vite C.H. (2003).

[114] Bauer J. Evaluation and dietary considerations in idiopathic hyperlipidemia in dogs. J Am Vet Med Assoc. (1995) 206:1684–8. doi: 10.2460/javma.1995.206.11.1684 7782237

[115] Jung M Lee KM Im Y Seok SH Chung H Kim DY. Nicotinamide (niacin) supplement increases lipid metabolism and ROS-induced energy disruption in triple-negative breast cancer: potential for drug repositioning as an anti-tumor agent. Mol Oncol. (2022) 16:1795–815. doi: 10.1002/1878-0261.13209 PMC9067146 35278276

[116] Bourgin M Kepp O Kroemer G. Immunostimulatory effects of vitamin B5 improve anticancer immunotherapy. Taylor Francis;. (2022) p:2031500. doi: 10.1080/2162402X.2022.2031500 PMC8794238 35096488

[117] Bravo-San Pedro JM Sica V Madeo F Kroemer G. Acyl-CoA-binding protein (ACBP): the elusive ‘hunger factor’linking autophagy to food intake. Cell Stress. (2019) 3:312. doi: 10.15698/cst 31656948 PMC6789435

[118] Trefely S Lovell CD Snyder NW Wellen KE. Compartmentalised acyl-CoA metabolism and roles in chromatin regulation. Mol Metab. (2020) 38:100941. doi: 10.1016/j.molmet.2020.01.005 32199817 PMC7300382

[119] López-Otín C Kroemer G. Hallmarks of health. Cell. (2021) 184:33–63. doi: 10.1016/j.cell.2020.11.034 33340459

[120] Deng P Valentino T Flythe MD Moseley HN Leachman JR Morris AJ. Untargeted stable isotope probing of the gut microbiota metabolome using 13C-labeled dietary fibers. J Proteome Res. (2021) 20:2904–13. doi: 10.1021/acs.jproteome.1c00124 PMC8784920 33830777

[121] Spencer CN McQuade JL Gopalakrishnan V McCulloch JA Vetizou M Cogdill AP. Dietary fiber and probiotics influence the gut microbiome and melanoma immunotherapy response. Science. (2021) 374:1632–40. doi: 10.1126/science.aaz7015 PMC8970537 34941392

[122] Nitto T Onodera K. The linkage between coenzyme a metabolism and inflammation: roles of pantetheinase. J Pharmacol Sci. (2013) 123:1–8. doi: 10.1254/jphs.13R01CP 23978960

[123] Berruyer C Martin F Castellano R Macone A Malergue F Garrido-Urbani S. Vanin-1–/– mice exhibit a glutathione-mediated tissue resistance to oxidative stress. Mol Cell Biol. (2004) 24:7214–24. doi: 10.1128/MCB.24.16.7214-7224.2004 PMC479710 15282320

[124] Berruyer C Pouyet L Millet V Martin FM LeGoffic A Canonici A. Vanin-1 licenses inflammatory mediator production by gut epithelial cells and controls colitis by antagonizing peroxisome proliferator-activated receptor γ activity. J Exp Med. (2006) 203:2817–27. doi: 10.1084/jem.20061640 PMC2118186 17145956

[125] Paul MS Saibil SD Han S Israni-Winger K Lien SC Laister RC. Coenzyme A fuels T cell anti-tumor immunity. Cell Metab. (2021) 33:2415–27. e6. doi: 10.1016/j.cmet.2021.11.010 34879240

[126] Giacalone S Spigariolo CB Bortoluzzi P Nazzaro G. Oral nicotinamide: The role in skin cancer chemoprevention. Dermatol Ther. (2021) 34:e14892. doi: 10.1111/dth.14892 33595161

[127] Galler A Tran J Krammer-Lukas S Höller U Thalhammer J Zentek J. Blood vitamin levels in dogs with Malignancies and the influence of chemotherapy. Wiener Tierärztliche Monatsschrift. (2015) 102:144–54.

[128] Parra M Stahl S Hellmann H. Vitamin B6 and its role in cell metabolism and physiology. Cells. (2018) 7:84. doi: 10.3390/cells7070084 30037155 PMC6071262

[129] Komatsu S-i Watanabe H Oka T Tsuge H Nii H Kato N. Vitamin B-6–supplemented diets compared with a low vitamin B-6 diet suppress azoxymethane-induced colon tumorigenesis in mice by reducing cell proliferation. J Nutr. (2001) 131:2204–7. doi: 10.1093/jn/131.8.2204 11481418

[130] He C Wang D Shukla SK Hu T Thakur R Fu X. Vitamin B6 competition in the tumor microenvironment hampers antitumor functions of NK cells. Cancer Discovery. (2023) 14:176–93. doi: 10.1158/2159-8290.c.7022625 PMC10784745 37931287

[131] Gylling B Myte R Schneede J Hallmans G Häggström J Johansson I. Vitamin B-6 and colorectal cancer risk: a prospective population-based study using 3 distinct plasma markers of vitamin B-6 status. Am J Clin Nutr. (2017) 105:897–904. doi: 10.3945/ajcn.116.139337 28275126

[132] Ames BN Wakimoto P. Are vitamin and mineral deficiencies a major cancer risk? Nat Rev Cancer. (2002) 2:694–704. doi: 10.1038/nrc886 12209158

[133] Merigliano C Mascolo E Burla R Saggio I Vernì F. The relationship between vitamin B6, diabetes and cancer. Front Genet. (2018) 9:388. doi: 10.3389/fgene.2018.00388 30271425 PMC6146109

[134] Noto H Tsujimoto T Sasazuki T Noda M. Significantly increased risk of cancer in patients with diabetes mellitus: a systematic review and meta-analysis. Endocr Practice. (2011) 17:616–28. doi: 10.4158/EP10357.RA 21454235

[135] Okada M Shibuya M Yamamoto E Murakami Y. Effect of diabetes on vitamin B6 requirement in experimental animals. Diab Obes Metab. (1999) 1:221–5. doi: 10.1046/j.1463-1326.1999.00028.x 11228757

[136] Rains JL Jain SK. Oxidative stress, insulin signaling, and diabetes. Free Radical Biol Med. (2011) 50:567–75. doi: 10.1016/j.freeradbiomed.2010.12.006 PMC3557825 21163346

[137] Blasiak J Arabski M Krupa R Wozniak K Zadrozny M Kasznicki J. DNA damage and repair in type 2 diabetes mellitus. Mutat Research/Fundamental Mol Mech Mutagen. (2004) 554:297–304. doi: 10.1016/j.mrfmmm.2004.05.011 15450427

[138] Goodarzi MT Navidi AA Rezaei M Babahmadi-Rezaei H. Oxidative damage to DNA and lipids: correlation with protein glycation in patients with type 1 diabetes. J Clin Lab anal. (2010) 24:72–6. doi: 10.1002/jcla.20328 PMC6647748 20333759

[139] Tatsch E Bochi GV Piva SJ De Carvalho JA Kober H Torbitz VD. Association between DNA strand breakage and oxidative, inflammatory and endothelial biomarkers in type 2 diabetes. Mutat Research/Fundamental Mol Mech Mutagen. (2012) 732:16–20. doi: 10.1016/j.mrfmmm.2012.01.004 22285873

[140] McKhann GM Mickelsen O Tower DB. Oxidative metabolism of incubated cerebral cortex slices from pyridoxine-deficient kittens. Am J Physiology-Legacy Content. (1961) 200:34–8. doi: 10.1152/ajplegacy.1961.200.1.34 13774046

[141] Bai SC Sampson DA Morris JG Rogers QR. Vitamin B-6 requirement of growing kittens. J Nutr. (1989) 119:1020–7. doi: 10.1093/jn/119.7.1020 2754508

[142] Blanchard PC Bai SC Rogers QR Morris JG. Pathology associated with vitamin B-6 deficiency in growing kittens. J Nutr. (1991) 121(11 Suppl):S77–8. doi: 10.1093/jn/121.suppl_11.S77 1834817

[143] Bai SC Sampson DA Morris JG Rogers QR. The level of dietary protein affects the vitamin B-6 requirement of cats. J Nutr. (1991) 121:1054–61. doi: 10.1093/jn/121.7.1054 2051225

[144] Buckmaster PS Holliday TA Bai SC Rogers QR. Brainstem auditory evoked potential interwave intervals are prolonged in vitamin B-6-deficient cats. J Nutr. (1993) 123:22–6. doi: 10.1093/jn/123.1.20 8421226

[145] Vail DM Chun R Thamm DH Garrett LD Cooley AJ Obradovich JE. Efficacy of pyridoxine to ameliorate the cutaneous toxicity associated with doxorubicin containing pegylated (Stealth) liposomes: a randomized, double-blind clinical trial using a canine model. Clin Cancer Res. (1998) 4:1567–71. 9626479

[146] Hymes J Wolf B. Biotinidase and its roles in biotin metabolism. Clinica chimica Acta. (1996) 255:1–11. doi: 10.1016/0009-8981(96)06396-6 8930409

[147] Chauhan J Dakshinamurti K. Transcriptional regulation of the glucokinase gene by biotin in starved rats. J Biol Chem. (1991) 266:10035–8. doi: 10.1016/S0021-9258(18)99181-7 2037560

[148] Peterson CT Rodionov DA Osterman AL Peterson SN. and their role in immune regulation and cancer. Nutrients. (2020) 12:3380. doi: 10.3390/nu12113380 33158037 PMC7693142

[149] Agrawal S Agrawal A Said HM. Biotin deficiency enhances the inflammatory response of human dendritic cells. Am J Physiology-Cell Physiol. (2016) 311:C386–C91. doi: 10.1152/ajpcell.00141.2016 PMC5129763 27413170

[150] Sghaier R Zarrouk A Nury T Badreddine I O’Brien N Mackrill JJ. Biotin attenuation of oxidative stress, mitochondrial dysfunction, lipid metabolism alteration and 7β-hydroxycholesterol-induced cell death in 158N murine oligodendrocytes. Free Radical Res. (2019) 53:535–61. doi: 10.1080/10715762.2019.1612891 31039616

[151] Järvinen E Ismail K Muniandy M Bogl L Heinonen S Tummers M. Biotin-dependent functions in adiposity: a study of monozygotic twin pairs. Int J Obes. (2016) 40:788–95. doi: 10.1038/ijo.2015.237 26601567

[152] Kuroishi T. Regulation of immunological and inflammatory functions by biotin. Can J Physiol Pharmacol. (2015) 93:1091–6. doi: 10.1139/cjpp-2014-0460 26168302

[153] Raza A Singh A Amin S Spallholz JE Sharma AK. Identification and biotin receptor-mediated activity of a novel seleno-biotin compound that inhibits viability of and induces apoptosis in ovarian cancer cells. Chemico-Biol Interact. (2022) 365:110071. doi: 10.1016/j.cbi.2022.110071 35921948

[154] Tripathi R Guglani A Ghorpade R Wang B. Biotin conjugates in targeted drug delivery: is it mediated by a biotin transporter, a yet to be identified receptor, or (an) other unknown mechanism (s)? J Enzyme Inhibition Med Chem. (2023) 38:2276663. doi: 10.1080/14756366.2023.2276663 PMC10653662 37955285

[155] Russell-Jones G McTavish K McEwan J Rice J Nowotnik D. Vitamin-mediated targeting as a potential mechanism to increase drug uptake by tumours. J inorganic Biochem. (2004) 98:1625–33. doi: 10.1016/j.jinorgbio.2004.07.009 15458825

[156] Russell-Jones G McEwan J. Amplification of biotin-mediated targeting. USA: Google Patents (2006).

[157] Rayan M Shadafny S Falah A Falah M Abu-Lafi S Asli S. A novel docetaxel-biotin chemical conjugate for prostate cancer treatment. Molecules. (2022) 27:961. doi: 10.3390/molecules27030961 35164226 PMC8839329

[158] Kundu BK Pragti Carlton Ranjith WA Shankar U Kannan RR Mobin SM. Cancer-targeted chitosan–biotin-conjugated mesoporous silica nanoparticles as carriers of zinc complexes to achieve enhanced chemotherapy in vitro and in *vivo* . ACS Appl Bio Mater. (2021) 5:190–204. doi: 10.1021/acsabm.1c01041 35014809

[159] Chen Y Meng W Chen M Zhang L Chen M Chen X. Biotin-decorated hollow gold nanoshells for dual-modal imaging-guided NIR-II photothermal and radiosensitizing therapy toward breast cancer. J Mater Chem B. (2023) 11:10003–18. doi: 10.1039/D3TB01736B 37843459

[160] Nogueira SP Brunetto MA Jeremias JT Gomes M Teshima E Carciofi AC. Dermatose responsiva à biotina em cão. Ciec Rural. (2010) 40:682–5. doi: 10.1590/S0103-84782010000300032

[161] Pieroth R Paver S Day S Lammersfeld C. Folate and its impact on cancer risk. Curr Nutr Rep. (2018) 7:70–84. doi: 10.1007/s13668-018-0237-y 30099693 PMC6132377

[162] Liew S-C. Folic acid and diseases-supplement it or not? Rev da Associacao Med Bras. (2016) 62:90–100. doi: 10.1590/1806-9282.62.01.90 27008500

[163] Rai V. Folate pathway gene MTHFR C677T polymorphism and risk of lung cancer in Asian populations. Asian Pacific J Cancer Prev. (2014) 15:9259–64. doi: 10.7314/APJCP.2014.15.21.9259 25422209

[164] Courtemanche C Elson-Schwab I Mashiyama ST Kerry N Ames BN. Folate deficiency inhibits the proliferation of primary human CD8+ T lymphocytes in vitro. J Immunol. (2004) 173:3186–92. doi: 10.4049/jimmunol.173.5.3186 15322179

[165] Courtemanche C Huang AC Elson-Schwab I Kerry N Ng BY Ames BN. Folate deficiency and ionizing radiation cause DNA breaks in primary human lymphocytes: a comparison. FASEB J. (2004) 18:209–11. doi: 10.1096/fj.03-0382fje 14597554

[166] Taştekin D Ertürk K Bozbey HU Ölmüşçelik O Kızıltan HŞ Tuna S. Plasma homocysteine, folate and vitamin B12 levels in patients with lung cancer. Exp Oncol. (2015) 37:218–22. doi: 10.31768/2312-8852.2015.37(3):218-222 26422108

[167] Mikkelsen K Prakash MD Kuol N Nurgali K Stojanovska L Apostolopoulos V. Anti-tumor effects of vitamin B2, B6 and B9 in promonocytic lymphoma cells. Int J Mol Sci. (2019) 20:3763. doi: 10.3390/ijms20153763 31374832 PMC6696026

[168] Miller JW Beresford SA Neuhouser ML Cheng T-YD Song X Brown EC. Homocysteine, cysteine, and risk of incident colorectal cancer in the Women’s Health Initiative observational cohort. Am J Clin Nutr. (2013) 97:827–34. doi: 10.3945/ajcn.112.049932 PMC3607656 23426034

[169] Galluzzi L Vacchelli E Michels J Garcia P Kepp O Senovilla L. Effects of vitamin B6 metabolism on oncogenesis, tumor progression and therapeutic responses. Oncogene. (2013) 32:4995–5004. doi: 10.1038/onc.2012.623 23334322

[170] Khanna R Karki K Pande D Negi R Khanna R. Inflammation, free radical damage, oxidative stress and cancer. Interdiscip J Microinflamm. (2014) 1:1000109. doi: 10.18632/oncotarget.17922

[171] Xue S Hu M Li P Ma J Xie L Teng F. Relationship between expression of PD-L1 and tumor angiogenesis, proliferation, and invasion in glioma. Oncotarget. (2017) 8:49702. doi: 10.18632/oncotarget.v8i30 28591697 PMC5564800

[172] Chanput W Peters V Wichers H. THP-1 and U937 Cells. In: The Impact of Food Bioactives on gut Health. Cham, Switzerland, Springer (2015). 29787063

[173] Shklar G Cataldo E Fitzgerald AL. The effect of methotrexate on chemical carcinogenesis of hamster buccal pouch. Cancer Res. (1966) 26:2218–24. 5921494

[174] Barich LL Schwarz J Barich D. Oral methotrexate in mice: a co-carcinogenic as well as an anti-tumor agent to methylcholanthrene-induced cutaneous tumors. J Invest Dermatol. (1962) 39:615–8. doi: 10.1038/jid.1962.160

[175] Baggott JE Vaughn WH Juliana MM Eto I Krumdieck CL Grubbs CJ. Effects of folate deficiency and supplementation on methylnitrosourea-induced rat mammary tumors. JNCI: J Natl Cancer Instit. (1992) 84:1740–4. doi: 10.1093/jnci/84.22.1740 1433358

[176] Al-Rubaie AZ Al-Jadaan SA Abd Al-Wahed AT Raadah IA. Synthesis, characterization and biological studies of some new organometallic compounds containing mercury, selenium and tellurium based on p-aminobenzoic acid. J Phys: Conf Ser. (2021). doi: 10.1088/1742-6596/2063/1/012003

[177] Cismesia AP Nicholls GR Polfer NC. Amine vs. carboxylic acid protonation in ortho-, meta-, and para-aminobenzoic acid: An IRMPD spectroscopy study. J Mol Spectrosc. (2017) 332:79–85. doi: 10.1016/j.jms.2016.10.020 28439142 PMC5400370

[178] Bousis S Setyawati I Diamanti E Slotboom DJ Hirsch AK. Energy-coupling factor transporters as novel antimicrobial targets. Adv Ther. (2019) 2:1800066. doi: 10.1002/adtp.201800066

[179] Maki T Takeda K. Benzoic acid and derivatives. In: Maki T Takeda K, (editors). Ullmann’s encyclopedia of industrial chemistry. vol. 3. Wiley-VCH Verlag GmbH & Co. (2000).

[180] Akberova S. New biological properties of p-aminobenzoic acid. Biol Bull Russian Acad Sci. (2002) 29:390–3. doi: 10.1023/A:1016871219882 12180014

[181] Xavier S MacDonald S Roth J Caunt M Akalu A Morais D. The vitamin-like dietary supplement para-aminobenzoic acid enhances the antitumor activity of ionizing radiation. Int J Radiat Oncol Biol Phys. (2006) 65:517–27. doi: 10.1016/j.ijrobp.2006.01.010 16690434

[182] Patel HM Bhardwaj V Sharma P Noolvi MN Lohan S Bansal S. Quinoxaline-PABA bipartite hybrid derivatization approach: Design and search for antimicrobial agents. J Mol Struct. (2019) 1184:562–8. doi: 10.1016/j.molstruc.2019.02.074

[183] Markitantova YV Akberova S Ryabtseva A Stroeva O. The effect of para-aminobenzoic acid on apoptosis processes in the adult rat conjunctiva and corneal epithelium in *vivo* after Hypobaric Hypoxia. Biol Bullet. (2018) 45:226–34. doi: 10.1134/S1062359018020061

[184] Crisan ME Bourosh P Maffei ME Forni A Pieraccini S Sironi M. Synthesis, crystal structure and biological activity of 2-hydroxyethylammonium salt of p-aminobenzoic acid. PloS One. (2014) 9:2:e10189. doi: 10.1371/journal.pone.0101892 PMC4108362 25054237

[185] Kadhum WR Oshizaka T Ichiro H Todo H Sugibayashi K. Usefulness of liquid–crystal oral formulations to enhance the bioavailability and skin tissue targeting of p-amino benzoic acid as a model compound. Eur J Pharm Sci. (2016) 88:282–90. doi: 10.1016/j.ejps.2016.04.003 27072433

[186] Afifi AM Saad AM Al-Husseini MJ Elmehrath AO Northfelt DW Sonbol MB. Causes of death after breast cancer diagnosis: A US population-based analysis. Cancer. (2020) 126:1559–67. doi: 10.1002/cncr.32648 31840240

[187] Ghanem NM Farouk F George RF Abbas SE El-Badry OM. Design and synthesis of novel imidazo [4, 5-b] pyridine based compounds as potent anticancer agents with CDK9 inhibitory activity. Bioorg Chem. (2018) 80:565–76. doi: 10.1016/j.bioorg.2018.07.006 30025343

[188] Salem IM Mostafa SM Salama I El-Sabbagh OI Hegazy WA Ibrahim TS. Design, synthesis and antitumor evaluation of novel pyrazolo [3, 4-d] pyrimidines incorporating different amino acid conjugates as potential DHFR inhibitors. J Enzyme Inhibition Med Chem. (2023) 38:203–15. doi: 10.1080/14756366.2022.2142786 PMC9673804 36382444

[189] Abuelizz HA Awad HM Marzouk M Nasr FA Bakheit AH Naglah AM. Exploiting the 4-Hydrazinobenzoic acid moiety for the development of anticancer agents: Synthesis and biological profile. Bioorg Chem. (2020) 102:104098. doi: 10.1016/j.bioorg.2020.104098 32702510

[190] Batt R Mann L. Specificity of the BT-PABA test for the diagnosis of exocrine pancreatic insufficiency in the dog. Vet Rec. (1981) 108:303–7. doi: 10.1136/vr.108.14.303 6972120

[191] Balluz LS Kieszak SM Philen RM Mulinare J. Vitamin and mineral supplement use in the United States: results from the third National Health and Nutrition Examination Survey. Arch Family Med. (2000) 9:258. doi: 10.1001/archfami.9.3.258 10728113

[192] Munteanu C Uţiu I Roşioru C Lang C. Chronic administration of red bull affects blood parameters in rats. Studia Universitatis Babeş-Bolyai, Biologia (2014) 59(2):89–98.

[193] Gianantonio KE. Primary Care: Dietary Supplement Use Among Patients and Implementation of Patient Supplement Education. USA: Kent State University, College of Nursing (2021).

[194] Bauer JA Frye G Bahr A Gieg J Brofman P. Anti-tumor effects of nitrosylcobalamin against spontaneous tumors in dogs. Investig New Drugs. (2010) 28:694–702. doi: 10.1007/s10637-009-9282-0 19557306

[195] Löffler M Carrey E Zameitat E. Orotate (orotic acid): An essential and versatile molecule. Nucleosides Nucleotides Nucleic Acids. (2016) 35:566–77. doi: 10.1080/15257770.2016.1147580 27906623

[196] Chiara F Allegra S Mula J Puccinelli MP Abbadessa G Mengozzi G. The strange case of orotic acid: the different expression of pyrimidines biosynthesis in healthy males and females. J Personal Med. (2023) 13:1443. doi: 10.3390/jpm13101443 PMC10608620 37888054

[197] Karachentsev YI Kravchun N Chernyaeva A Dunaeva I Kholodny A Efimenko T. Place of magnesium orotate in the complex therapy of patients with type 2 diabetes mellitus with hyperuricemia. Problems Endocr Pathol. (2020) 71:23–9. doi: 10.21856/j-PEP.2020.1.03

[198] Kalacheva A Gromova O Grishina T Bogacheva T Demidov V Torshin IY. Investigation of the effects of magnesium orotate in a model of primary generalized seizures. Neurol Neuropsych Psychosom. (2017) 9:61–6.

[199] Schiopu C Ştefănescu G Diaconescu S Bălan G Gimiga N Rusu E. Magnesium orotate and the microbiome-gut-brain axis modulation: new approaches in psychological comorbidities of gastrointestinal functional disorders. Nutrients. (2022) 14:1567. doi: 10.3390/nu14081567 35458129 PMC9029938

[200] Löffler M Carrey EA Zameitat E. Orotic acid, more than just an intermediate of pyrimidine *de novo* synthesis. J Genet Genomics. (2015) 42:207–19. doi: 10.1016/j.jgg.2015.04.001 26059769

[201] Dagmar S Veronika H Katrin S Hans N Erich EF. Studies on the chemical identity and biological functions of pangamic acid. Arzneimittelforschung. (1999) 49:335–43. doi: 10.1055/s-0031-1300424 10337453

[202] Stacpoole PW. The pharmacology of dichloroacetate. Metabolism. (1989) 38:1124–44. doi: 10.1016/0026-0495(89)90051-6 2554095

[203] Herbert V. Pangamic acid (“vitamin B15”). Am J Clin Nutr. (1979) 32:1534–40. doi: 10.1093/ajcn/32.7.1534 377937

[204] Stacpoole P Harwood H Varnado C. Regulation of rat liver hydroxymethylglutaryl coenzyme A reductase by a new class of noncompetitive inhibitors. Effects of dichloroacetate and related carboxylic acids on enzyme activity. J Clin Invest. (1983) 72:1575–85. doi: 10.1172/JCI111116 PMC370445 6630519

[205] Stacpoole PW Moore GW Kornhauser DM. Metabolic effects of dichloroacetate in patients with diabetes mellitus and hyperlipoproteinemia. New Engl J Med. (1978) 298:526–30. doi: 10.1056/NEJM197803092981002 625308

[206] Moore GW Swift LL Rabinowitz D Crofford OB Oates JA Stacpoole PW. Reduction of serum cholesterol in two patients with homozygous familial hypercholesterolemia by dichloroacetate. Atherosclerosis. (1979) 33:285–93. doi: 10.1016/0021-9150(79)90180-1 486225

[207] Rastopchin I. Effect of calcium pangamate on the cholesterol index of atherogenicity in cerebral arteriosclerosis patients. Zhurnal Nevropatol i Psikhiatrii Imeni SS Korsakova (Moscow Russia: 1952). (1984) 84:1020–3. 6475410

[208] Savula M Kravchenko N Poznanski A. Chemotherapy of destructive pulmonary tuberculosis with antioxidants and antihypoxic agents. Problemy Tuberkuleza. (1993) 5):18–20. 8295877

[209] Ames BN McCann J Yamasaki E. Methods for detecting carcinogens and mutagens with the Salmonella/mammalian-microsome mutagenicity test. Mutat Res;(Netherlands). (1975) 31:347–64. doi: 10.1016/0165-1161(75)90046-1 768755

[210] Colman N Herbert V Gardner A Gelernt M. Mutagenicity of dimethylglycine when mixed with nitrite: possible significance in human use of pangamates. Proc Soc Exp Biol Med. (1980) 164:9–12. doi: 10.3181/00379727-164-40815 6154952

[211] Stacpoole PW. Toxicity of chronic dichloroacetate. N Engl J Med. (1979) 300:372. doi: 10.1056/NEJM197902153000726 215910

[212] Herbert V Gardner A Colman N. Mutagenicity of dichloroacetate, an ingredient of some formulations of pangamic acid (trade-named “vitamin B15”). Am J Clin Nutr. (1980) 33:1179–82. doi: 10.1093/ajcn/33.6.1179 6992558

[213] Young VR Newberne PM. Vitamins and cancer prevention: issues and dilemmas. Cancer. (1981) 47:1226–40. doi: 10.1002/(ISSN)1097-0142 7237379

[214] Marshall F Adamson R Long J. Some pharmacologic properties of pangamic acid (vitamin B-15). Proc Soc Exp Biol Med. (1961) 107:420–2. doi: 10.3181/00379727-107-26644 13767203

[215] Askar MA El-Sayyad GS Guida MS Khalifa E Shabana ES Abdelrahman IY. Amygdalin-folic acid-nanoparticles inhibit the proliferation of breast cancer and enhance the effect of radiotherapy through the modulation of tumor-promoting factors/immunosuppressive modulators in vitro. BMC Complement Med Therapies. (2023) 23:162. doi: 10.1186/s12906-023-03986-x PMC10199568 37210478

[216] Lehmane H Kohonou AN Tchogou AP Ba R Dah-Nouvlessounon D Didagbé O. Antioxidant, anti-inflammatory, and anti-cancer properties of amygdalin extracted from three cassava varieties cultivated in Benin. Molecules. (2023) 28:4548. doi: 10.3390/molecules28114548 37299029 PMC10254302

[217] Barakat H Aljutaily T Almujaydil MS Algheshairy RM Alhomaid RM Almutairi AS. Amygdalin: a review on its characteristics, antioxidant potential, gastrointestinal microbiota intervention, anticancer therapeutic and mechanisms, toxicity, and encapsulation. Biomolecules. (2022) 12:1514. doi: 10.3390/biom12101514 36291723 PMC9599719

[218] Erikel E Yuzbasioglu D Unal F. A study on Amygdalin’s genotoxicological safety and modulatory activity in human peripheral lymphocytes in *vitro* . Environ Mol Mutagen. (2023) 64:291–308. doi: 10.1002/em.22543 37161892

[219] Shi J Chen Q Xu M Xia Q Zheng T Teng J. Recent updates and future perspectives about amygdalin as a potential anticancer agent: A review. Cancer Med. (2019) 8:3004–11. doi: 10.1002/cam4.2197 PMC6558459 31066207

[220] Lee HM Moon A. Amygdalin regulates apoptosis and adhesion in Hs578T triple-negative breast cancer cells. Biomol Ther. (2016) 24:62. doi: 10.4062/biomolther.2015.172 PMC4703354 26759703

[221] Zhang Q Liu J Zhang M Wei S Li R Gao Y. Apoptosis induction of fibroblast-like synoviocytes is an important molecular-mechanism for herbal medicine along with its active components in treating rheumatoid arthritis. Biomolecules. (2019) 9:795. doi: 10.3390/biom9120795 31795133 PMC6995542

[222] Chen Y Ma J Wang F Hu J Cui A Wei C. Amygdalin induces apoptosis in human cervical cancer cell line HeLa cells. Immunopharmacol immunotoxicol. (2013) 35:43–51. doi: 10.3109/08923973.2012.738688 23137229

[223] Aamazadeh F Ostadrahimi A Rahbar Saadat Y Barar J. Bitter apricot ethanolic extract induces apoptosis through increasing expression of Bax/Bcl-2 ratio and caspase-3 in PANC-1 pancreatic cancer cells. Mol Biol Rep. (2020) 47:1895–904. doi: 10.1007/s11033-020-05286-w 32026321

[224] Aydın D Özkan K Aydın A. The combination of amygdalin with some anticancer, antiparasitic, and antigout drugs against MG63, Saos2, SW1353, and FL cells in vitro. J Med Food. (2021) 24:1230–4. doi: 10.1089/jmf.2020.0143 33733877

[225] Chang H-K Shin M-S Yang H-Y Lee J-W Kim Y-S Lee M-H. Amygdalin induces apoptosis through regulation of Bax and Bcl-2 expressions in human DU145 and LNCaP prostate cancer cells. Biol Pharm Bullet. (2006) 29:1597–602. doi: 10.1248/bpb.29.1597 16880611

[226] Chen Y Al-Ghamdi AA Elshikh MS Shah MH Al-Dosary MA Abbasi AM. Phytochemical profiling, antioxidant and HepG2 cancer cells’ antiproliferation potential in the kernels of apricot cultivars. Saudi J Biol Sci. (2020) 27:163–72. doi: 10.1016/j.sjbs.2019.06.013 PMC6933278 31889831

[227] Mosayyebi B Mohammadi L Kalantary-Charvadeh A Rahmati M. Amygdalin decreases adhesion and migration of MDA-MB-231 and MCF-7 breast cancer cell lines. Curr Mol Pharmacol. (2021) 14:667–75. doi: 10.2174/1874467213666200810141251 32778045

[228] Cecarini V Selmi S Cuccioloni M Gong C Bonfili L Zheng Y. Targeting proteolysis with cyanogenic glycoside amygdalin induces apoptosis in breast cancer cells. Molecules. (2022) 27:7591. doi: 10.3390/molecules27217591 36364419 PMC9657530

[229] Moradipoodeh B Jamalan M Zeinali M Fereidoonnezhad M Mohammadzadeh G. Specific targeting of HER2-positive human breast carcinoma SK-BR-3 cells by amygdaline-ZHER2 affibody conjugate. Mol Biol Rep. (2020) 47:7139–51. doi: 10.1007/s11033-020-05782-z 32929653

[230] Dimitrov M Iliev I Bardarov K Georgieva D Todorova T. Phytochemical characterization and biological activity of apricot kernels’ extract in yeast-cell based tests and hepatocellular and colorectal carcinoma cell lines. J Ethnopharmacol. (2021) 279:114333. doi: 10.1016/j.jep.2021.114333 34146630

[231] Park H-J Yoon S-H Han L-S Zheng L-T Jung K-H Uhm Y-K. Amygdalin inhibits genes related to cell cycle in SNU-C4 human colon cancer cells. World J gastroenterol: WJG. (2005) 11:5156. doi: 10.3748/wjg.v11.i33.5156 16127745 PMC4320388

[232] Cassiem W de Kock M. The anti-proliferative effect of apricot and peach kernel extracts on human colon cancer cells in vitro. BMC complement Altern Med. (2019) 19:1–12. doi: 10.1186/s12906-019-2437-4 30696432 PMC6352493

[233] Hosny S Sahyon H Youssef M Negm A. Prunus Armeniaca L. seed extract and its amygdalin containing fraction induced mitochondrial-mediated apoptosis and autophagy in liver carcinogenesis. Anti-Cancer Agents Med Chem (Formerly Curr Med Chemistry-Anti-Cancer Agents). (2021) 21:621–9. doi: 10.2174/1871520620666200608124003 32510292

[234] Mamdouh AM Khodeer DM Tantawy MA Moustafa YM. *In-vitro* and *in-vivo* investigation of amygdalin, metformin, and combination of both against doxorubicin on hepatocellular carcinoma. Life Sci. (2021) 285:119961. doi: 10.1016/j.lfs.2021.119961 34536497

[235] El-Desouky MA Fahmi AA Abdelkader IY Nasraldin KM. Anticancer effect of amygdalin (vitamin B-17) on hepatocellular carcinoma cell line (HepG2) in the presence and absence of zinc. Anti-Cancer Agents Med Chem (Formerly Curr Med Chemistry-Anti-Cancer Agents). (2020) 20:486–94. doi: 10.2174/1871520620666200120095525 31958042

[236] Salama RH Ramadan A Alsanory T Herdan M Fathallah O Alsanory A. Experimental and therapeutic trials of amygdalin. Int J Biochem Pharmacol. (2019) 1:21–6. doi: 10.18689/ijbp

[237] Lo M Ling V Wang Y Gout P. The xc– cystine/glutamate antiporter: a mediator of pancreatic cancer growth with a role in drug resistance. Br J cancer. (2008) 99:464–72. doi: 10.1038/sj.bjc.6604485 PMC2527809 18648370

[238] Fan J Ye J Kamphorst JJ Shlomi T Thompson CB Rabinowitz JD. Quantitative flux analysis reveals folate-dependent NADPH production. Nature. (2014) 510:298–302. doi: 10.1038/nature13236 24805240 PMC4104482

[239] Maddocks OD Athineos D Cheung EC Lee P Zhang T Van Den Broek NJ. Modulating the therapeutic response of tumours to dietary serine and glycine starvation. Nature. (2017) 544:372–6. doi: 10.1038/nature22056 28425994

[240] Wu Z Wei D Gao W Xu Y Hu Z Ma Z. TPO-induced metabolic reprogramming drives liver metastasis of colorectal cancer CD110+ tumor-initiating cells. Cell Stem Cell. (2015) 17:47–59. doi: 10.1016/j.stem.2015.05.016 26140605

[241] Munteanu C Achim L Muresan R Souca M Prifti E. The relationship between circadian rhythm and cancer disease. Int J Mol Sci. (2024) 25:5846. doi: 10.3390/ijms25115846 38892035 PMC11172077

